# Endometrium-derived mesenchymal stem cells suppress progression of endometrial cancer via the DKK1-Wnt/β-catenin signaling pathway

**DOI:** 10.1186/s13287-023-03387-4

**Published:** 2023-06-07

**Authors:** Yuhui Xu, Jiali Hu, Qiaoying Lv, Chenyi Shi, Mengdi Qiu, Liying Xie, Wei Liu, Bingyi Yang, Weiwei Shan, Yali Cheng, Bing Zhao, Xiaojun Chen

**Affiliations:** 1 Obstetrics and Gynecology Hospital of Fudan University, 419 Fangxie Road, Shanghai, 200011 People’s Republic of China; 2 Shanghai Key Laboratory of Female Reproductive Endocrine Related Diseases, Fudan University, Shanghai, 200011 People’s Republic of China; 3 State Key Laboratory of Genetic Engineering, School of Life Sciences, Human Phenome Institute, Fudan University, Shanghai, 200438 People’s Republic of China

**Keywords:** eMSCs, Endometrial cancer, Wnt/β-catenin signaling, DKK1

## Abstract

**Background:**

Mesenchymal stem cell (MSC) therapy is an attractive treatment option for various cancers. Whether MSCs can be used to treat well-differentiated endometrial cancer (EC) remains unclear. The aim of this study is to explore the potential therapeutic effects of MSCs on EC and the underlying mechanisms.

**Methods:**

The effects of adipose-derived MSCs (AD-MSCs), umbilical-cord-derived MSCs (UC-MSCs), and endometrium-derived MSCs (eMSCs) on the malignant behaviors of EC cells were explored via in vitro and in vivo experiments. Three EC models, including patient-derived EC organoid lines, EC cell lines, and EC xenograft model in female BALB/C nude mice, were used for this study. The effects of MSCs on EC cell proliferation, apoptosis, migration, and the growth of xenograft tumors were evaluated. The potential mechanisms by which eMSCs inhibit EC cell proliferation and stemness were explored by regulating DKK1 expression in eMSCs or Wnt signaling in EC cells.

**Results:**

Our results showed that eMSCs had the highest inhibitory effect on EC cell viability, and EC xenograft tumor growth in mice compared to AD-MSCs and UC-MSCs. Conditioned medium (CM) obtained from eMSCs significantly suppressed the sphere-forming ability and stemness-related gene expression of EC cells. In comparison to AD-MSCs and UC-MSCs, eMSCs had the highest level of Dickkopf-related protein 1 (DKK1) secretion. Mechanistically, eMSCs inhibited Wnt/β-catenin signaling in EC cells via secretion of DKK1, and eMSCs suppressed EC cell viability and stemness through DKK1-Wnt/β-catenin signaling. Additionally, the combination of eMSCs and medroxyprogesterone acetate (MPA) significantly inhibited the viability of EC organoids and EC cells compared with eMSCs or MPA alone.

**Conclusions:**

The eMSCs, but not AD-MSCs or UC-MSCs, could suppress the malignant behaviors of EC both in vivo and in vitro via inhibiting the Wnt/β-catenin signaling pathway by secreting DKK1. The combination of eMSCs and MPA effectively inhibited EC growth, indicating that eMSCs may potentially be a new therapeutic strategy for young EC patients desiring for fertility preservation.

**Supplementary Information:**

The online version contains supplementary material available at 10.1186/s13287-023-03387-4.

## Introduction

Endometrial cancer (EC) is one of the most common gynecological malignancies [1]. With the modern changes of lifestyle and diet, EC has shown increased incidence yearly and younger trend [2]. Approximately 5% of EC patients are under the age of 40, among whom half are childless at the time of diagnosis [3, 4]. This makes fertility-preserving treatment an important need for patients. Currently, high-dose progestin therapy is the first-line treatment for patients with early-stage well-differentiated endometrioid endometrial cancer (EEC) [5]. However, progestin therapy is associated with relatively low complete response (CR) rates, long treatment duration, and undesirable side effects such as weight gain, risk of thrombosis, and endometrial damage. In accordance with a recent systematic meta-analysis, 15%–25% of patients with endometrial atypical hyperplasia (the precancerous stage of EC) and 25%–50% of EC patients do not achieve CR after 6–7 months of progestin treatment [6]. Therefore, new strategies are needed to improve the efficacy of fertility-preserving treatment in patients with early EC.

Mesenchymal stem cells (MSCs) are multipotent stem cells with self-renewal and multi-lineage-differentiation potential [7]. Previous studies have indicated that MSCs have anti-tumor effects and might be useful for the treatment of cancers including breast, gastric, and ovarian cancers [8–10]. It has been reported that bone-marrow-derived MSCs (BM-MSCs) decreased the number and size of colorectal tumors in a tumor xenograft animal model [8], and placenta-derived MSCs combined with sorafenib effectively inhibited the progression of hepatocellular carcinoma both in vitro and in vivo [9]. MSCs can be obtained from multiple sources, such as the bone marrow, umbilical cord, adipose tissues, and endometrium, and MSCs exert different anti-tumor effects depending on the tissue origin. It has been reported that adipose-derived MSCs (AD-MSCs) promoted the growth and vascularization of EC-cell-derived tumor xenograft in mice [11], whereas umbilical-cord-derived MSCs (UC-MSCs) inhibited EC cell proliferation and migration [12]. No study has examined the effect of endometrium-derived MSCs (eMSCs) on EC malignant behaviors.eMSCs might be an attractive option for EC treatment due to the following reasons. First, eMSCs can be easily isolated from endometrial tissues obtained from endometrial biopsy. Second, eMSCs have low potential to tumorigenesis or transform into malignant neoplasms when transplanted into endometrium in preclinical or clinical studies [13–15]. Third, eMSCs have been reported to be able to repair mechanically damaged endometria in patients with Asherman syndrome [16, 17]. However, there has been no reports on the efficacy and safety of eMSCs for the treatment of EC.

In this study, we evaluated the therapeutic potential of MSCs derived from three different tissues (the eMSCs, AD-MSCs, and UC-MSCs) on EC. The effects of MSCs on the malignant behaviors of EC cell lines, patient-derived EC organoid lines, and EC-cell-derived xenografts in female BALB/C nude mice were evaluated. The mechanisms by which eMSCs inhibited EC was also investigated. We found that eMSCs, but not AD-MSCs or UC-MSCs, could suppress malignant behaviors of EC both in vivo and in vitro*.* eMSCs suppressed EC growth through inhibition of the Wnt/β-catenin pathway via secretion of Dickkopf-related protein 1 (DKK1). This suggested that eMSCs might be a potential candidate for cell-based fertility-preserving treatment of EC.

## Materials and methods

### Collection of human tissue samples

Fresh tissues from patients receiving hysterectomy were obtained between November 2019 and May 2020 at the Obstetrics and Gynecology Hospital of Fudan University. Three well-differentiated endometrial endometrioid cancer (EEC, G1) tumor tissues were used to establish EC organoid lines. Three eMCSs (eMSCs-1, eMSCs-2 and eMSCs-3) were isolated from three different patients with leiomyoma or cervical intraepithelial neoplasia who received hysterectomy. All the three patients had normal endometrium confirmed by pathological evaluation. Omental adipose tissues were used to isolate AD-MSCs and umbilical cord tissues were used to isolate UC-MSCs. The patient information was reported in Additional file 1: Table S1 and S2.

### Culture and identification of MSCs

Human AD-MSCs, UC-MSCs, and eMSCs were isolated as reported previously [18–20]. MSCs were cultured in DMEM/F12 (HyClone, USA) with 10% fetal bovine serum (FBS), 100 U/mL penicillin, and 0.1 mg/mL streptomycin (all from Gibco, USA). MSCs were passaged every 4–6 days, and MSCs at passages 5–8 were used for cell-based assays. Identification of AD-MSCs, UC-MSCs and eMSCs were performed and seen in Additional file 8: Supplementary Materials and Methods.

### EC organoid lines and culture

**Fig. 1:**
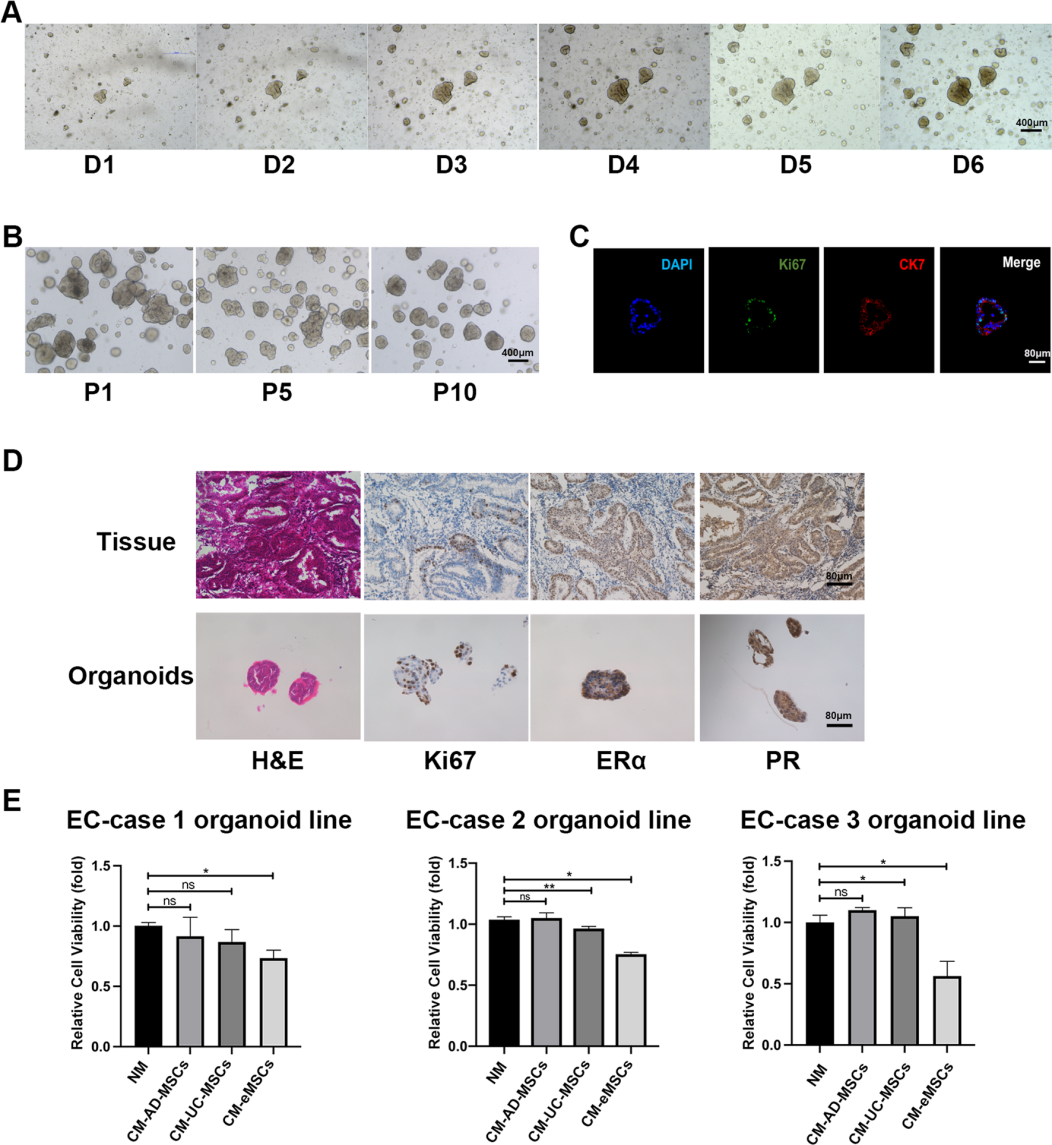
eMSCs inhibited the viability of EC patient-derived organoids. **A** Bright field images of EC organoids were taken from day 1 to day 6 after establishment. Original magnification, 4 × ; Scale bar, 400 μm. **B** Bright field images of EC organoids at Passage 1, Passage 5, and Passage 10. Original magnification, 4 × ; Scale bar, 400 μm. **C** Immunofluorescence staining images for DAPI, Ki67, and cytokeratin 7 (CK7) in EC organoids. Original magnification, 20 × ; Scale bar, 80 μm. **D** H&E and IHC staining images for Ki67, ERα, and PR in EC tissues and organoids. Original magnification, 20 × ; Scale bar, 80 μm. **E** eMSCs had the strongest inhibitory effect on the viability of EC organoids. EC organoids were treated with NM or CM derived from AD-MSCs, UC-MSCs, or eMSCs respectively for 96 h before three-dimensional cell viability assay. Three organoid lines derived from three patients with well-differentiated endometrial endometrioid cancer were used. Each EC organoid line was tested independently and was repeated three times in the same condition with independent analysis. NM, normal medium; CM, conditioned medium; Data were analyzed by ratio *t*-test (**E**). ns, not significant; *P < 0.05; **P < 0.01; ***P < 0.001

EC cells were isolated from fresh EC tissues as described previously [21]. Three organoid lines derived from three patients with well-differentiated endometrial endometrioid cancer were used in Figs. 1E and 7B-C. Basic information of these three patients was shown in Additional file 1: Table S1. Each EC organoid line was tested independently and was repeated three times in the same condition with independent analysis. Unless otherwise stated, EC-case 2 organoid line was used in all organoid-associated experiments. As for EC organoid culture, dissociated epithelial cells were resuspended in Matrigel and pipetted into a 24-well-plate (20 μL per well) with 400 μL medium in each well. The culture plate was incubated for 15 min at 37 °C, and then 400 μL culture medium was added to each well. The organoids were then cultured at 37 °C with 5% CO_2_ and monitored daily to assess spheroid formation. The culture medium was changed every 3 days and organoids were passaged every 7 days.

The culture medium for EC organoids contained 1 × Advanced DMEM/F12, 1 × B27 supplement, 1 × Glutamax, 100 U/mL penicillin, 0.1 mg/mL streptomycin, 5 ng/mL EGF, 250 ng/mL R-spondin-1,100 ng/mL Noggin, 500 nM A83-01, 5 mM Y-27632, and 5 mM nicotinamide [21].

### Cell lines and culture

The well-differentiated EC cell line RL95-2 was kindly provided by Dr. Yu Yinhua (MD Anderson Cancer Center, Houston, TX, USA), and HEC-1A was kindly provided by Dr. Wei Lihui (Peking University People’s Hospital, Beijing, China). Both cell lines were cultured in DMEM/F12 medium with 10% FBS.

### Drug intervention

The following drugs were used for cell interventions as described in each experiment: medroxyprogesterone acetate (MPA) (Sigma Aldrich, USA), DKK1 (Sino Biological, China), CHIR-99021 (Selleck, USA), anti-DKK1-neutralizing antibody (R&D System, USA), and Foscenvivint (Selleck, USA).

### Conditioned medium (CM) collection and enzyme linked immunosorbent assay (ELISA)

MSCs were seeded into 100-mm culture dishes at approximately 4 × 10^5^ cells and cultured for 24 h. When density of MSCs reached 60%-70% confluency, the supernatant was discarded and complete medium (DMEM/F12 medium with 10% FBS) was added to culture the MSCs. After 24 h, the supernatant was collected and called conditioned medium (CM). For CM collection after transfection of siRNAs that targeted DKK1 and its siMock in eMSCs, siRNAs were transfected when density of eMSCs reached 30%-40% confluency, and complete medium (DMEM/F12 medium with 10% FBS) was replaced after 24 h of transfection. After another 24 h, CM was collected. The CM was then centrifugated (1,000 rpm for 15 min) and filtered using a 0.22-micron syringe filter (Sigma Aldrich, USA) to remove cells and debris for the following study. The DKK1 levels of CM obtained from the three MSCs were evaluated using an ELISA kit (Quantikine, R&D Systems) according to the manufacturer’s instructions.

### Cell viability assay

EC cells were seeded at 3000 cells per well into 96-well plates and treated with indicated medium or drugs for 48 h and then measured by Cell Counting Kit-8 (CCK-8, Dojindo, Japan). EC cells were seeded at 1000 cells per well into 96-well plates and treated with CM of MSCs for 0–7 days (growth curve) and then measured by CCK-8. Then CCK-8 was used to measure EC cell proliferation, as per the manufacturer’s instructions. EC organoids were seeded at 1000 cells per well into 96-well plates and treated with indicated medium or drugs. Then CellTiter-Glo® three-dimensional Cell Viability Assay (Promega, USA) was used to measure the viability of EC organoids. And the detailed information for the results of cell viability assay was provided in the Additional file 9: ‘raw data of cell viability experiments’.

### Migration assay

EC cells (2 × 10^5^ per well) were seeded in the upper chamber of Transwell plates with 8-μm pore size membranes (Corning) in 24-well plates; MSCs (5 × 10^4^ per well) were seeded in the lower chambers. After 24 h of incubation, migrated cells at the back of the upper chambers were stained with 0.1% crystal violet and counted under a microscope.

### Sphere formation assay

Sphere formation assay was performed as described previously [22, 23]. Cells were digested into a single cell suspension, resuspended in Matrigel at 3 × 10^3^ cells per 20 μL Matrigel and then seeded into a 12-well-plate. The culture plate was placed in an incubator at 37 °C for 10 min, and 1 mL medium was added per well. The numbers of visible spheres were counted on day 15.

### siRNA transfection

siRNA sequences that targeted DKK1 and siMock were listed in Additional file 1: Table S3. Transfection of eMSCs with siMock or siRNAs that targeted DKK1 was performed using Lipofectamine 3000 (ThermoFisher, USA) by following the manufacturer’s instructions.

### Immunofluorescence (IF) staining and immunohistochemical (IHC) staining

IF and IHC staining were performed as described previously [24, 25]. Primary antibodies used for IHC and IF staining were as follows: cytokeratin 7 (1:200), anti-estrogen receptor α (ERα) (1:200), anti-Progesterone receptor (PR) (1:200), anti-Ki67 (1:200), anti-β-catenin (1:200), and anti-DKK1 (1:200) (all from Cell Signaling Technology, USA). Secondary fluorescent-dye-tagged antibody were anti-mouse Alexa Fluor® 488 or anti-rabbit Alexa Fluor® 647, both from Life Technology.

### Western blotting

Western blotting analysis was performed as described previously [23]. Primary antibodies used were as follows: anti-β-catenin (1:1000), anti-C-MYC (1:1000), anti-AXIN2 (1:1000), anti-DKK1 (1:1000), anti-Nanog (1:1000), anti-BMI1 (1:1000), anti-BAX (1:1000), anti-BCL2 (1:1000), and anti-GAPDH (1:5000) (all from Abcam).

### RNA extraction and quantificational real-time polymerase chain reaction (qRT-PCR)

Total RNAs extracted from EC cells were reverse transcribed into cDNAs using the Reverse Transcription System (Promega, USA), following the manufacturer’s instructions. qRT-PCR was performed using SYBR Green Real-time PCR Master Mix (Takara, Japan). PCR primers were designed on the basis of cDNA sequences from the NCBI database. Primer sequences used for qRT-PCR were listed in Additional file 1: Table S4. The expression of target genes relative to GAPDH was calculated with 2^–ΔΔCt^ method, where ΔCt = Ct target gene–Ct GAPDH, and ΔΔCt = ΔCt sample group–ΔCt control group. All the tests were performed in triplicate.

### Subcutaneous EC mouse models

Female BALB/C nude mice (3–4 weeks old) were obtained from Shanghai Laboratory Animal Center (Shanghai, China). Mice were maintained in a specific pathogen free (SPF) and temperature-controlled facility on a 12-h light/darkness cycle. After 1 week of feeding, the random number was generated using SPSS software (version 18.0) and 28 mice with similar age and weight were divided into 4 groups accordingly: RL95-2 or RL95-2 cells plus MSCs (n = 7 per group). The mice were subcutaneously injected with RL95-2 cells, alone (2 × 10^6^) or with MSCs (4 × 10^5^), at the right scapular regions. Tumor weight was the primary outcome in this study. At 28 days after the tumor cell injection, the animals were euthanized, and the tumors were harvested for analyses. All experimental procedures were approved by the Institutional Animal Care and Use Committee of Fudan University.

### Statistical analysis

Data are shown as the mean ± standard deviation (SD) of triplicate experiments. Unpaired *t*-test and ratio *t*-test were used for statistical analysis by Graphpad Prism 8.0. A two-tailed P-value of less than 0.05 was considered statistically significant.

## Results

### eMSCs inhibited the viability of EC patient-derived organoids

We first asked whether MSCs originated from different tissues had the same effects on the viability of well-differentiated EC. Organoids derived from EC patients were successfully established to mimic the characteristics of EC in vivo. The organoids formed 24 h after culturing, with a gradual increase in diameter from day 1 to day 6 (Fig. 1A). Organoids were stably cultured for more than 10 passages (Fig. 1B). IF staining of cytokeratin 7 in EC organoids confirmed their epithelial origin (Fig. 1C). H&E staining of the organoids and their original tumors, as well as IHC staining of ERα, PR, and Ki67 demonstrated that the organoids and their original tumor had similar histological characteristics (Fig. 1D). These EC organoids were then used in the following experiments.

The three types of MSCs (AD-MSCs, UC-MSCs, and eMSCs) were successfully isolated from the adipose tissues, umbilical cord, and normal endometrium. MSC markers CD73, CD90, and CD105 was positively expressed, and CD45 was negatively expressed (Additional file 2: Fig. S1A). Besides, in vitro differentiation assays of the three MSCs were performed and the results showed that all three MSCs had multi-differentiation capabilities, including adipogenic, osteogenic, and chondrogenic differentiation (Additional file 2: Fig. S1B). The findings identified the properties of MSCs used in this study. In this study, we used MSCs from passages 5 to 8, and flow cytometry analysis showed positive expression of MSC markers CD73, CD90, and CD105 in eMSCs at passage 8 (Additional file 2: Fig. S 1C), suggesting that eMSCs at passage 8 maintain the property of stemness. Then immunomodulatory potential assay was performed to evaluate the immunomodulatory ability of the three MSCs. Results showed that CM obtained from eMSCs had little effect on the proportion of CD4^+^/CD3^+^T cells and CD8^+^/CD3^+^T cells, while CM from AD-MSCs and UC-MSCs decreased the proportion of CD4^+^/CD3^+^T cells and CD8^+^/CD3^+^T cells in the LPS-stimulated condition (Additional file 3: Fig. S2A-B). Cytokine and cytolytic granule production of CD8^+^ T cells remained unchanged in the CM from eMSCs group in the LPS-stimulated condition, while CM from AD-MSCs slightly decreased cytolytic granule production, and CM from UC-MSCs slightly increased TNF-α and cytolytic granule production (Additional file 3: Fig. S2C-E).

EC organoids were cultured in CM (derived from the three MSCs types) for 96 h before the three-dimensional cell viability assay was performed. The results showed that CM from eMSCs significantly decreased the cell viability in three EC organoid lines, compared to those from AD-MSCs and UC-MSCs (Fig. 1E). These results indicated that eMSCs had the highest inhibitory effect on EC viability, among the three types of MSCs.

### eMSCs suppressed the malignant behaviors of EC cells

**Fig. 2:**
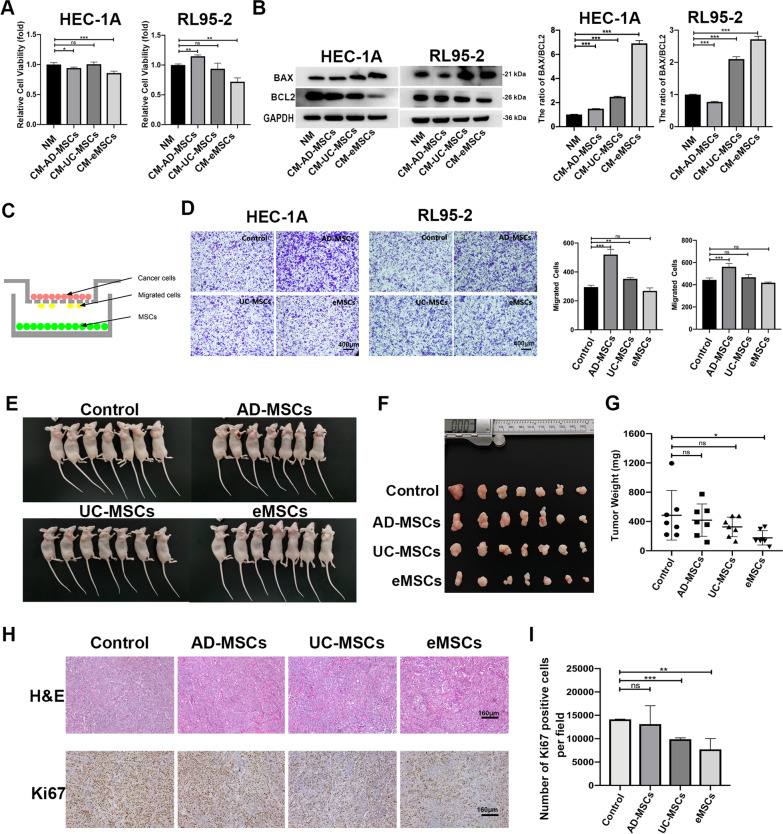
eMSCs suppressed the malignant behaviors of EC cells. **A** CM derived from eMSCs showed the most significant anti-proliferative effect on EC cells, compared to AD-MSCs and UC-MSCs. RL95-2 and HEC-1A cells were treated with NM or CM derived from MSCs for 48 h before Cell Viability Assay. **B** CM derived from eMSCs markedly increased BAX expression and BAX/BCL2 ratio and decreased BCL2 expression in EC cells, compared to AD-MSCs and UC-MSCs. RL95-2 and HEC-1A cells were treated with NM or CM derived from MSCs for 48 h before western blotting. BAX/BCL2 ratio was calculated from densitometry by ImageJ. **C** RL95-2 and HEC-1A cells seeded in 24‐well chambers were co-cultured with or without MSCs, as shown in the pattern diagram for 24 h before cell migration assay. **D** eMSCs did not affect EC cell migration, while AD-MSCs promoted EC cell migration. UC-MSCs slightly induced migration of HEC-1A cells but had no effect on RL95-2 cells. Bright field images were taken (Left), and the numbers of migratory cells per bright field image were calculated (Right). Original magnification 4 × ; Scale bar, 400 μm. **E–G** eMSCs had the highest inhibitory effect on EC xenograft tumor growth, compared to AD-MSCs and UC-MSCs. RL952 cells alone, or RL95-2 cells and MSCs (5:1) were subcutaneously injected in female nude mice. Mice were sacrificed after 28 days (E), tumor tissues were photographed **(F),** and tumor weight was then measured **(G)**. **H-I** H&E and IHC staining images for Ki67 in xenograft tumors. NM, normal medium; CM, conditioned medium; The blots of BAX, BCL2, and GAPDH were all cropped **(B)** and full-length blots were presented in Additional file 7: Fig. S6. Data were analyzed by ratio *t*-test (**A**) and unpaired *t*-test (**B,** **D,** **G**, and **I**). ns, not significant; *P < 0.05; **P < 0.01; ***P < 0.001

To confirm the inhibitory effect of eMSCs on EC, we evaluated the effect of the three MSCs types on the malignant behaviors of EC in vitro using EC cell lines HEC-1A and RL95-2*.* Cell proliferation assays showed that only CM derived from eMSCs significantly inhibited EC cell proliferation, whereas no or less inhibitory effect was seen with CM derived from AD-MSCs or UC-MSCs (Fig. 2A). To further confirm the effect of MSCs on EC cell growth, CM from the three MSCs with different proportion (0%, 10%, 30% and 100%) was used to treat EC cells for 48 h (Additional file 4: Fig. S3A). With the increase of CM proportion, the inhibitory effect of CM from eMSCs on EC cells gradually and significantly increased. Thus, for the growth curve analysis, HEC-1A cells were treated with 100% CM from different MSCs. The results showed that eMSCs had the highest inhibitory effect on EC viability especially from day 4 to 7 (Additional file 4: Fig. S3B). These findings suggested that eMSCs did have a significant inhibitory effect on EC cell growth. Next, the effect of MSCs on EC cell apoptosis was evaluated by examining the expression levels of BAX and BCL2 (two critical regulators of apoptosis) and the BAX/BCL2 ratio. The CM from eMSCs had the most potent ability to promote EC cell apoptosis, as demonstrated by the increases of BAX protein expression and BAX/BCL2 ratio in EC cells (Fig. 2B). The migration assay of EC cells induced by MSCs were performed. As the cell culture time was 24 h in the migration assay and the EC cell growth was not affected significantly by CM of MSCs treatment at 24 h (Additional file 4: Fig. S3B), we suggest that the changes of migrated EC cells were mainly due to MSCs-mediated migration effect but not the proliferation effect. Results showed that AD-MSCs stimulated the migration of HEC-1A and RL95-2 cells, whereas UC-MSCs slightly induced the migration of HEC-1A cells but had no effect on RL95-2 cells. The eMSCs did not affect the migration of EC cells (Fig. 2C-D). These in vitro data showed that eMSCs inhibited proliferation, promoted apoptosis, and had no effect on the migration of EC cells.

**Fig. 3:**
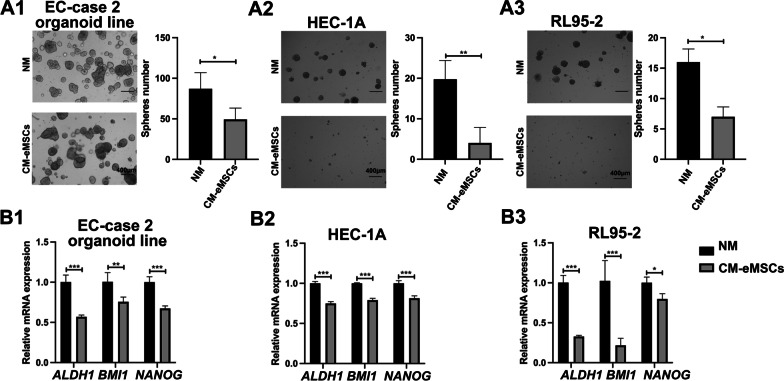
eMSCs suppressed the tumor-related stemness of EC cells. **A1-A3** eMSCs inhibited sphere-forming ability of EC organoids and cell lines. Single cell suspensions of EC cells were seeded in Matrigel and treated with NM or CM from eMSCs for 15 days. The numbers of visible spheres were calculated. Original magnification, 4 × ; Scale bar, 400 μm. **B1-B3** eMSCs inhibited the transcriptional levels of EC stemness-associated genes (*ALDH1*, *BMI1*, *and NANOG*) both in EC organoids and cell lines. CM derived from eMSCs was used to treat EC organoids for 96 h or EC cell lines for 48 h. EC cells were then harvested for qRT-PCR analysis. NM, normal medium; CM, conditioned medium; Data were analyzed by unpaired* t*-test (**A-B**). *P < 0.05; **P < 0.01; ***P < 0.001

To investigate the effects of MSCs on tumor growth in vivo, a subcutaneous EC model was established in female nude mice using RL95-2 cells, alone or combined with MSCs. The results showed that mice injected with RL95-2 cells and eMSCs had the lowest tumor weight, as compared to the control group and the groups injected with RL95-2 cells and AD-MSCs or UC-MSCs (Fig. 2E-G). Histological analyses showed that EC xenografts formed by RL95-2 cells and eMSCs had the lowest Ki67 expression, when compared to the other groups (Fig. 2H-I). It is known that MSCs affect cancer progression by regulating the niches of cancer stem cells [26]. We then asked whether eMSCs inhibited the stemness of EC cells. The number of visible spheres formed, stemness-related genes (*ALDH1, BMI1*, and *NANOG*) of EC organoids and EC cell lines both were significantly suppressed by CM derived from eMSCs, according to respective sphere formation assays and qRT-PCR (Fig. 3A-B). These results suggested that eMSCs inhibited the stemness of EC cells. Taken together, our findings demonstrated that eMSCs had the best ability to inhibit the malignant behaviors of EC cells.

### eMSCs inhibited EC cell proliferation and stemness through paracrine secretion of DKK1

**Fig. 4:**
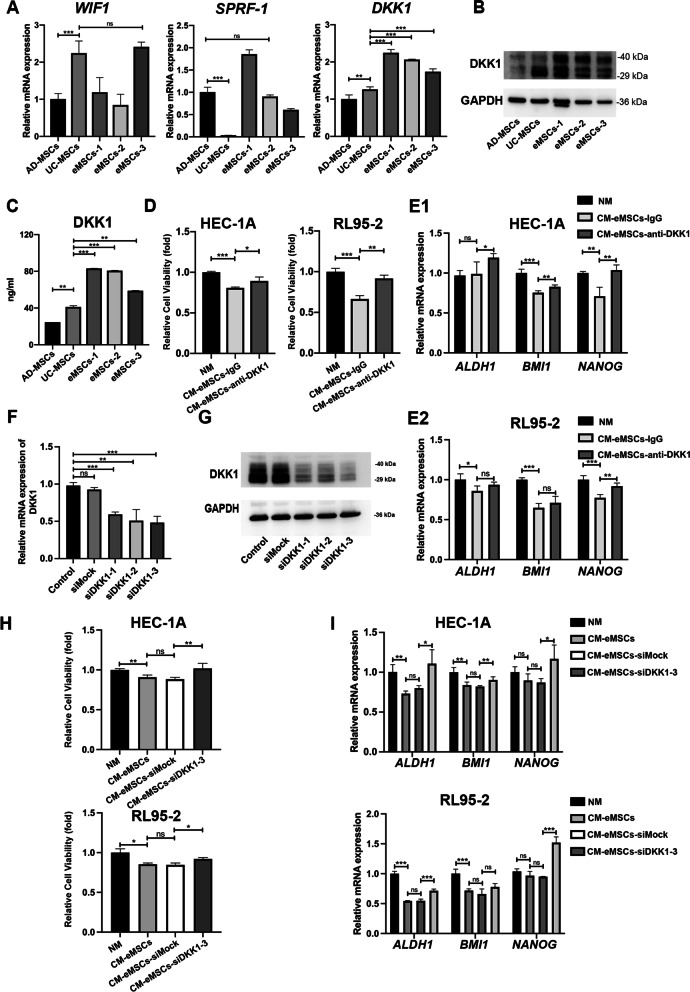
eMSCs inhibited EC cell proliferation and stemness through paracrine secretion of DKK1. **A** eMSCs showed the highest level of DKK1 mRNA, compared to AD-MSCs and UC-MSCs, while the mRNA levels of SFRP1 and WIF1 were not significantly elevated in eMSCs, compared to other MSCs. **B** eMSCs showed the highest level of DKK1 in eMSCs cell, compared to AD-MSCs and UC-MSCs, as detected by western blotting. **C** eMSCs showed the highest level of DKK1 secretion compared to AD-MSCs and UC-MSCs. CM obtained from the three MSCs was collected under the same condition as shown in Materials and Methods and the DKK1 levels were evaluated using an ELISA kit. **D-E** DKK1-neutralizing antibody compromised the inhibitory effect of eMSCs on proliferation **(D)** and stemness **(E)** in EC cells. CM obtained from eMSCs was pre-treated with DKK1-neutralizing antibody (anti-DKK1) or isotype control (IgG) overnight and then used to treat EC cells for 48 h. EC cells were harvested for Cell Viability Assay **(D)** and qRT-PCR **(E)** respectively. **F–G.** The mRNA and protein levels of DKK1 were reduced in eMSCs by transfection of DKK1 siRNAs. eMSCs were transfected with three different siRNAs (siDKK1-1, -2, and -3) targeting DKK1 or siMock for 6 h. At 48 h after transfection, eMSCs were harvested for examination of mRNA and protein levels of DKK1. siDKK1-3 was used for further investigation. **H-I.** Silencing DKK1 in eMSCs abrogated the anti-proliferation and anti-stemness effects of eMSCs on EC cells. The CM of eMSCs was collected after 24 h of siDKK1-3 transfection. EC cells were treated with the indicated CM of eMSCs for 48 h and then the mRNA levels of stemness-associated genes, *ALDH1*, *BMI1*, *and NANOG*, were measured by qRT-PCR. NM, normal medium; CM, conditioned medium; The blots of DKK1 and GAPDH were all cropped (**B** **and** **G**) and full-length blots were presented in Additional file 7: Figure S6. Data were analyzed by unpaired *t*-test (**A,** **C,** **E,** **F,** **I**) and ratio *t*-test (**D** and **H**). ns, not significant; *P < 0.05; **P < 0.01; ***P < 0.001

We further investigated the potential mechanisms by which eMSCs inhibit EC. Because the CM from eMSCs inhibited EC cell stemness, we hypothesized that factors secreted by eMSCs may contribute to the inhibitory effect of eMSCs on EC cells. DKK1, SFRP1, and WIF1 are important factors secreted by MSCs, and these have been reported to inhibit the Wnt signaling pathway [27]. Therefore, we examined the three types of MSCs for these three factors. qRT-PCR showed that *DKK1* mRNA was highly expressed in eMSCs, in comparison to AD-MSCs and UC-MSCs (Fig. 4A), and highest DKK1 expression in eMSCs was confirmed by western blots (Fig. 4B). ELISA results showed the highest level of DKK1 in culture supernatants from eMSCs, compared to those from AD-MSCs and UC-MSCs (Fig. 4C). Whether DKK1 mediated eMSCs-induced EC cell proliferation and stemness was further investigated in the following study.

To confirm the effect of DKK1 on EC cells, CM of eMSCs was pre-incubated with an anti-DKK1-neutralizing antibody before the treatment of EC cells. CCK-8 and qRT-PCR assays showed that the anti-proliferation and anti-stemness effects of eMSCs were compromised after DKK1 neutralization (Fig. 4D–E). This indicated that DKK1 secreted by eMSCs inhibited EC cell proliferation and stemness. To further confirm that DKK1 is an important factor secreted by eMSCs that inhibits the stemness of EC cells, three siRNAs targeting DKK1 were synthesized to silence DKK1 expression, as verified by qRT-PCR and western blotting (Fig. 4F–G). The decreased proliferation and stemness-related genes (*ALDH1*, *BMI1*, and *NANOG*) of EC cells were both rescued after silencing DKK1 expression in eMSCs (Fig. 4H–I). Furthermore, histological analyses showed that EC xenografts formed from RL95-2 cells and eMSCs had the higher DKK1 expression, compared to the control group (Additional file 5: Fig. S4A). Taken together, these data suggested that DKK1 is an important protein secreted by eMSCs, and it inhibits the stemness of EC cells.

### eMSCs inhibited Wnt/β-catenin signaling in EC cells through DKK1

**Fig. 5:**
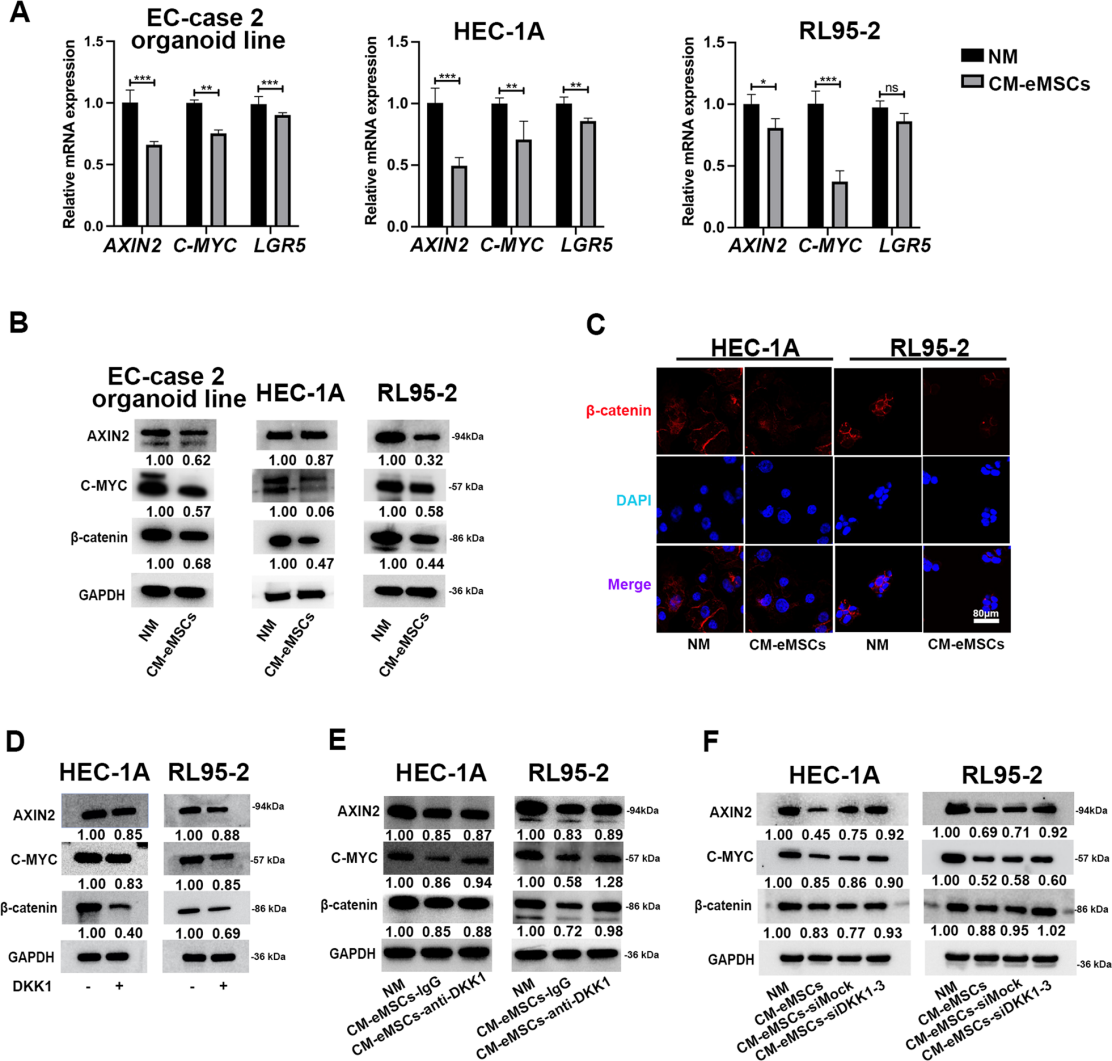
eMSCs inhibited Wnt/β-catenin signaling in EC cells through DKK1. **A,** **B** eMSCs inhibited expression of β-catenin protein and Wnt target genes in EC organoids and cell lines. CM from eMSCs was used to treat EC organoids for 96 h or EC cell lines for 48 h. EC cells were then harvested for qRT-PCR and western blotting. EC-case 2 organoid line was used in Fig. 5A. **C** Expression of β-catenin protein was reduced in cytoplasm and nuclei of EC cells after treatment of CM derived from eMSCs. Original magnification, 40 × ; Scale bar, 80 μm. **D** DKK1 inhibited expression of β-catenin and Wnt target proteins (AXIN2 and C-MYC) in EC cell lines. EC cells were treated with DKK1 (100 ng/ml) for 48 h and then harvested for western blotting. **E** DKK1-neutralizing antibody compromised the inhibitory effect of eMSCs on Wnt signaling in EC cells. CM obtained from eMSCs was pre-treated with DKK1-neutralizing antibody (anti-DKK1) or isotype control (IgG) overnight and then used to treat EC cells for 48 h. EC cells were harvested for western blotting analysis. **F** Silencing DKK1 by siDKK1-3 rescued Wnt signaling, which was inhibited by eMSCs in EC cells. EC cells were treated with the indicated CM of eMSCs for 48 h. EC cells were harvested for western blotting analysis. NM, normal medium; CM, conditioned medium. The blots of AXIN2, C-MYC, β-catenin, and GAPDH were all cropped (**B,** **D-F**) and full-length blots were presented in Additional file 7: Figure S6. Data were analyzed by unpaired *t*-test (**A**). ns, not significant; *P < 0.05; **P < 0.01; ***P < 0.001

Previous studies have shown that the Wnt/β-catenin signaling pathway regulates the stemness of EC cells [28]. Both qRT-PCR and western blotting showed that CM of eMSCs down-regulated the expression of Wnt/β-catenin target genes in EC organoids and cell lines (Fig. 5A-B). Western blotting and IF staining showed that β-catenin was reduced by CM of eMSCs, especially in the cytoplasm of EC cells (Fig. 5B-C). Furthermore, histological analyses showed that EC xenografts formed by RL95-2 cells and eMSCs had the lower β-catenin expression, compared to the control group (Additional file 5: Fig. S4B). We then asked whether eMSCs inhibited Wnt/β-catenin signaling in EC cells through DKK1. DKK1 treatment alone inhibited the expression of β-catenin and Wnt target proteins (AXIN2 and C-MYC) in EC cell lines (Fig. 5D). To further confirm that DKK1 mediated eMSCs-induced inhibition of β-catenin signaling in EC cells, DKK1 level was downregulated by pre-incubating CM of eMSCs with an anti-DKK1 neutralizing antibody or by transfecting siDKK1-3 in eMSCs, before the collection of CM. We found that the expression of β-catenin and Wnt targets, C-MYC and AXIN2, were partially rescued in EC cells, after treatment of CM that were obtained by the above methods (Fig. 5E-F). Additionally, decreased proliferation of EC organoids induced by CM of eMSCs was rescued in CM of eMSCs-siDKK1 (Additional file 6: Fig. S5A-B). This suggested that DKK1 was an important factor that mediated eMSCs-induced Wnt/β-catenin signaling inhibition.

### eMSCs inhibited EC cell proliferation and stemness through DKK1-Wnt/β-catenin signaling

**Fig. 6:**
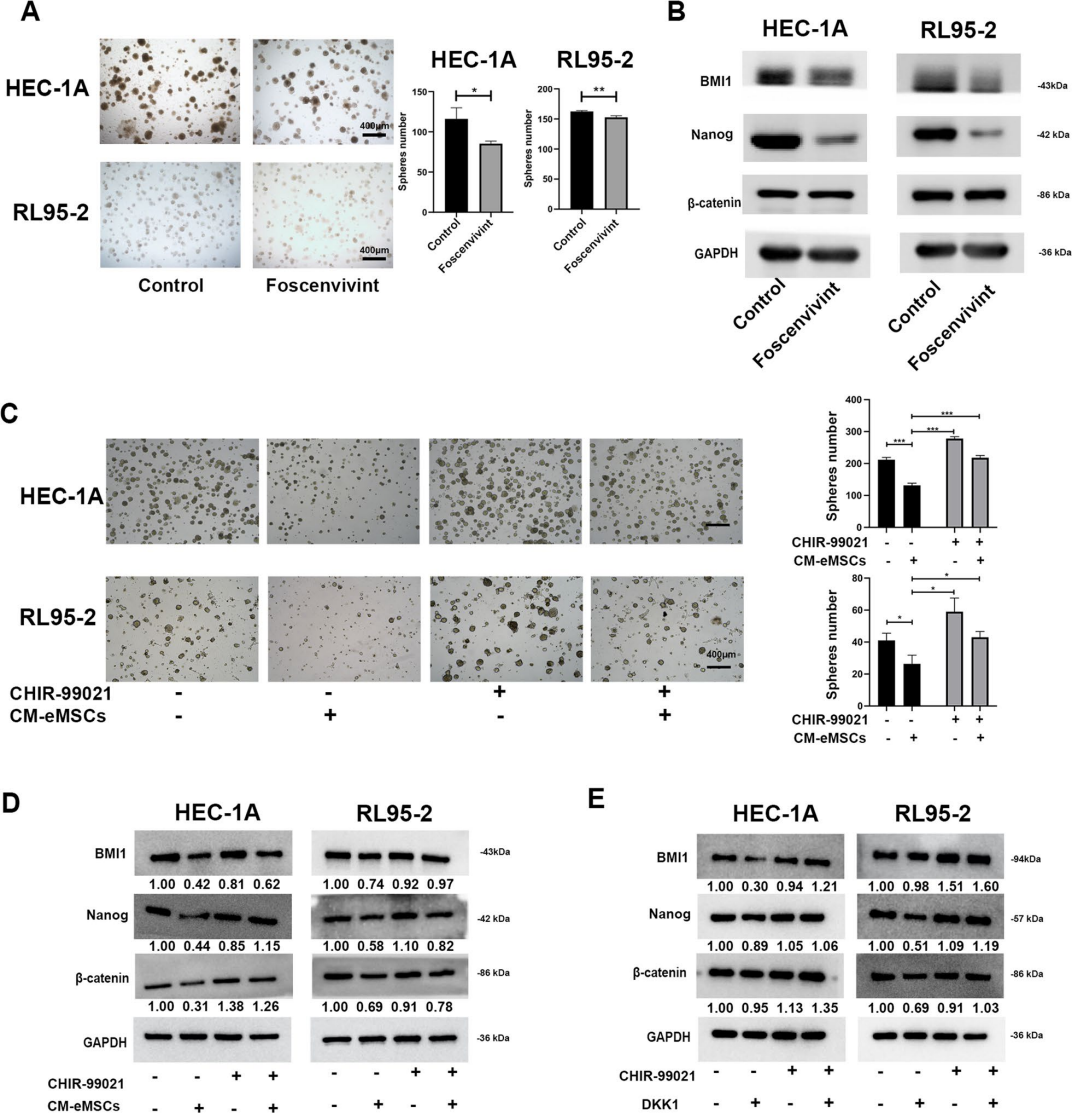
eMSCs inhibited EC cell proliferation and stemness through DKK1-Wnt/β-catenin signaling. **A** The inhibitory effect of Wnt inhibitor Foscenvivint on EC cell sphere-forming ability. EC cells were treated with or without Foscenvivint (0.5 μM) for 15 days. The numbers of visible spheres were calculated and compared. Original magnification, 4 × ; Scale bar, 400 μm. **B** The inhibitory effect of Foscenvivint on expression of stemness-related proteins in EC cells. EC cells were treated with or without Foscenvivint (2 μM) for 48 h and then were harvested to analyze β-catenin and stemness-related proteins by western blotting. **C** The inhibitory effect of CM derived from eMSCs on sphere-forming ability was reversed after activating Wnt signaling. EC cells were treated with or without Wnt activator CHIR-99021 (5 nM) in the presence or absence of CM derived from eMSCs for 15 days. The numbers of visible spheres were calculated and compared. Original magnification, 4 × ; Scale bar, 400 μm. **D** The inhibitory effect of CM derived from eMSCs on Wnt/β-catenin signaling and stemness-related proteins were reversed after activating Wnt signaling by CHIR-99021. EC cells were treated with or without Wnt activator CHIR-99021 (5 nM) in the presence or absence of CM derived from eMSCs for 48 h and then were harvested to analyze β-catenin and Wnt target proteins by western blotting. **E** CHIR-99021 treatment rescued DKK1-induced inhibition of Wnt/β-catenin signaling and stemness-related proteins. EC cells were treated with or without Wnt activator CHIR-99021 (5 nM) in the presence or absence DKK1 (DKK1) for 48 h and then were harvested to analyze β-catenin and stemness-related proteins by western blotting. CM, conditioned medium; The blots of BMI1, Nanog, β-catenin, and GAPDH were all cropped (**B,** **D,** **and** **E**) and full-length blots were presented in Additional file 7: Figure S6. Data were analyzed by unpaired *t*-test (**A,** **C**). ***P < 0.001

To further confirm that Wnt/β-catenin signaling mediated eMSCs- or DKK1- induced inhibition of EC cell proliferation and stemness, Foscenvivint, a Wnt inhibitor, was used to verify the inhibitory effect of Wnt pathway on EC cells. Result showed that Foscenvivint decreased sphere-forming ability and expression of stemness-related proteins in EC cells (Fig. 6A-B). This is consistent with the findings of eMSCs and DKK1. And then, CHIR-99021, an activator of Wnt signaling, was used. The results showed that CHIR-99021 pre-treatment restored the sphere-forming ability of EC cells, which was inhibited by eMSCs (Fig. 6C). Additionally, CHIR-99021 pre-treatment increased and restored the expression of β-catenin and stemness-related proteins (BMI1 and Nanog) in EC cells, which was inhibited respectively by eMSCs or DKK1 (Fig. 6D, E). This suggested that eMSCs inhibited EC cell proliferation and stemness through DKK1-Wnt/β-catenin signaling.

### eMSCs combined with MPA effectively inhibited EC viability

**Fig. 7:**
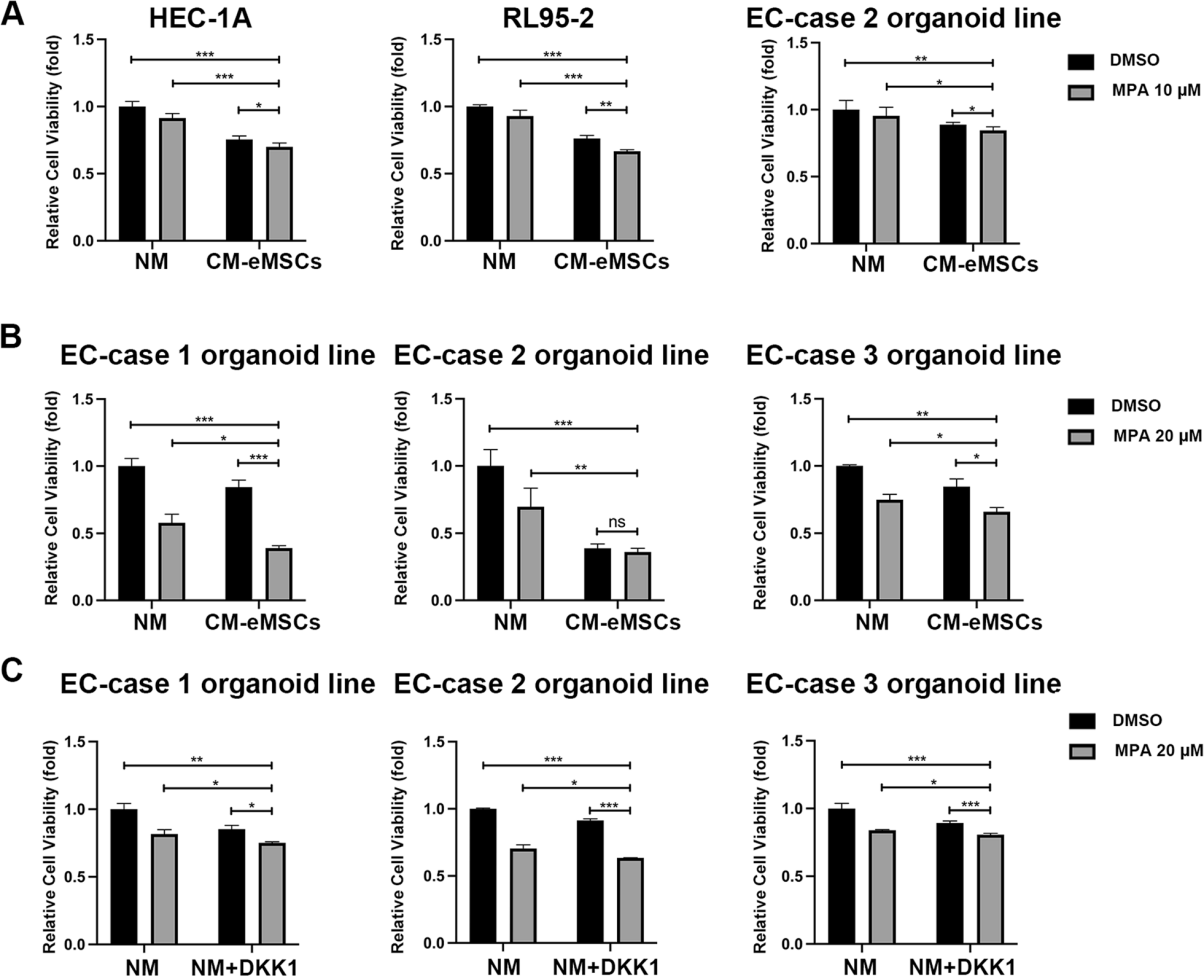
eMSCs combined with MPA had the highest inhibitory effect on EC growth. **A** CM obtained from eMSCs combined with MPA had the highest proliferation-inhibiting effect on EC cells. RL95-2 and HEC-1A cells were treated with 10 μM MPA with or without CM of eMSCs for 48 h before Cell Viability Assay. EC organoids were treated with 10 μM MPA with or without CM of eMSCs for 96 h before CCK-8 assay. **B** CM obtained from eMSCs combined with 20 μM MPA effectively inhibited viability of EC organoids, compared to using CM or MPA alone. Three-dimensional cell viability assay was used to assess the viability of EC organoids treated with MPA and/or CM of eMSCs for 96 h. **C** DKK1 combined with MPA had highest anti-proliferative effect on EC organoids compared with DKK1 or MPA alone. EC organoids were treated with or without MPA (20 μM) in the presence of DKK1 (100 ng/mL) or not for 96 h to assess cell viability by three-dimensional cell viability assay. NM, normal medium; CM, conditioned medium; Data were analyzed by ratio *t*-test (**A-C**). ns, not significant; *P < 0.05; **P < 0.01; ***P < 0.001

Our results showed that eMSCs effectively inhibited the malignant behaviors of EC cells, which indicated that eMSCs might be a potential cell-based therapy for EC patients who desire fertility-preserving treatment. High-dose progestin is the main regimen for the fertility-preserving treatment of EC. We therefore evaluated whether eMSCs improved the therapeutic effects of progestins on EC. EC cell lines and organoids were treated with MPA and/or CM derived from eMSCs. The combination of MPA and eMSCs had the strongest viability-inhibiting effect on both EC cell lines and organoids, compared to MPA or CM alone (Fig. 7A-B). As DKK1 was an important factor that mediated eMSCs-induced EC cell growth inhibition, DKK1 with or without MPA, were used to treat EC organoids. DKK1 combined with MPA significantly suppressed the proliferation of EC organoids compared with DKK1 or MPA alone (Fig. 7C). These preliminary results suggested that the combination of eMSCs and MPA might have a better therapeutic effect on young EC patients who desire fertility-preserving treatment.

## Discussion

In this study, we demonstrated that eMSCs effectively inhibited the proliferation and stemness of EC cells via the DKK1-Wnt/β-catenin signaling pathway*.* Additionally, we found that the combination of eMSCs and MPA had a better inhibitory effect on EC cell growth than any other treatment alone. The eMSCs are easy to obtain and cultivate, and they have been used for repairing mechanically damaged endometria and restoring endometrial fertility [29]. Therefore, we speculated that the combination of eMSCs and progestins might be a potential candidate for fertility-preserving treatment for EC patients.

The safety of eMSCs in treating EC is an important issue. Previous studies reported that transplantation of eMSCs into damaged endometrium could repair damaged endometrium without inducing carcinogenesis [13–15]. But no studies have shown whether eMSCs is safe for treating EC. In this study, we had designed a series of in vitro and in vivo experiments to verify the safety of eMSCs. Results showed that eMSCs suppressed the malignant behaviors of EC in EC cell lines, EC organoid lines and animal models with no immunosuppression. We did not evaluate whether long term co-culturing of eMSCs and EC cells could induce tumorigenesis of eMSCs in this study. More profound studies are needed to confirm the safety of eMSCs before it can be safely used in clinical settings.

Our study demonstrated that eMSCs might be a safe and effective treatment option for well-differentiated EC. The use of MSCs as a treatment for cancer has been controversial, due to their oncogenic and tumor-supporting potentials. Previous studies have reported that the oncoprotein c-Fos can induce mesenchymal cells to form cartilage or bone tumors [30]. MSCs can evolve into tumor-associated MSCs that promote cancer development. For example, bone-marrow-derived MSCs exhibit a tumor-promoting ability in osteosarcoma [31]. However, there has also been multiple reports that show MSCs could be used as an effective treatment for various tumors such as breast, gastric, and ovarian cancers [8–10]. In this study, through in vitro experiments, patient-EC-cell-derived organoids, and murine tumor xenograft model, we demonstrated that eMSCs had the most potent inhibitory effect on well-differentiated EC, compared to AD-MSCs and UC-MSCs, indicating that eMSCs might a safe and effective treatment option for well-differentiated EC. Tersoglio et al. also reported that sub-endometrial transplantation of eMSCs safely and effectively improved the endometrial generation and pregnancy rate in infertile women who had failed repeated embryo transplantation [15].

Our data showed that eMSCs inhibited both the proliferation and stemness of EC cells through DKK1-Wnt/β-catenin signaling. MSCs have been reported to inhibit tumor progression via various paracrine factors in hepatocellular carcinoma [32], breast cancer [33], ovarian cancer, and glioma [34], these factors include insulin-like growth factor-binding protein 3 [32], transforming growth factor-β [35] and DKK1 [32, 34, 36, 37]. It was reported that DKK1, secreted by MSCs, an inhibitor of the Wnt pathway, could inhibit tumor progression in various cancers. The AD-MSCs could inhibit lymphoblast proliferation [37], and UC-MSCs inhibited the growth of C6 glioma cells [34]; both act through the DKK1-Wnt/β-catenin signaling pathway. Classical Wnt/β-catenin signaling is an important regulator that maintains the self-renewal and differentiation of endometrial stem cells, and overactive Wnt/β-catenin signaling promotes EC malignancy [31]. Wang et al*.* reported that progestin-induced DKK1 and FOXO1 inhibited early endometrial carcinogenesis through Wnt/β-catenin signaling [38]. Our data showed for the first time that eMSCs secreted the highest level of DKK1, compared to AD-MSCs and UC-MSCs. We further proved the effective inhibitory effect of eMSCs on EC proliferation and stemness via paracrine secretion of DKK1, which was not found in AD-MSCs or UC-MSCs. Our result showed that DKK1 plays a vital role in the tumor-suppressive function of eMSCs. Exogenous DKK1 or small molecular canonical Wnt inhibitor can be considered for reinforcing treatment effect with progestins. However, these drugs should be given regularly and repeatedly. Compared with these drugs, with the advantage of self-renewal properties, eMSCs can secrete DKK1 stably and continuously once implanted into the endometrium. These make eMSCs also an attractive choice for enhancing MPA effect in the fertility-preserving treatment of EC. But more preclinical and clinical studies are needed to further confirm the safety and efficacy of eMSCs for the treatment of EC. For example, for promoting the potential applications of eMSCs in clinical treatment, standardized collection and culture procedures should be established and rigorous criteria should be used to evaluate the eMSCs. In the future, a good manufacturing practice-compatible and cost-effective protocol is needed to be presented for potential applications of eMSCs in clinical treatment.

Progestins is a first-line fertility-preserving treatment for EC patients [39]. Mechanistically, progestins act as an inhibitor of Wnt signaling in EC cells [38]. In our study, the combination of eMSCs with MPA had a better inhibitory effect on EC cells than eMSCs or MPA alone. We speculate that eMSCs and MPA may act together to inhibit EC cell stemness via Wnt signaling. Fertility-preserving regimens, which include high-dose oral progestins and repeated endometrial lesion resection, together with tumor-caused endometrial basal layer damage, might cause endometrial damage or intrauterine adhesions [40]. This could impair endometrial fecundity and result in low pregnancy rates. Previous studies have reported that endometrial mesenchymal/stromal cells could repair the damaged endometrium and improve pregnancy outcomes [17, 41, 42]. These reports and our data indicate that eMSCs combined with progestins might improve the outcome of fertility-preserving treatment in young EC patients. However, further study is needed to test this hypothesis.

This study is not without limitations. We primarily focused on the regulatory effects of eMSCs on EC cells. The regulatory effects of EC cells on eMSCs were not investigated, although our results from the mouse EC xenograft model showed a significantly inhibitory effect of eMSCs on EC growth. Additionally, the tumor microenvironment was not investigated in this study, and whether the tumor-associated immune microenvironment could shape eMSCs into tumor-promoting eMSCs was not evaluated. Further studies are needed to confirm the safety and efficacy of eMSCs for the treatment of EC.

## Conclusions

In summary, our study demonstrated that eMSCs suppressed EC cell growth and stemness via inhibiting the Wnt/β-catenin signaling pathway by secreting DKK1. Moreover, eMSCs combined with MPA had an increased anti-proliferative effect on EC cells, compared to using MPA or eMSCs alone. Our findings indicated that eMSCs may potentially be a new fertility-preserving strategy for well-differentiated EC. The safety and efficacy of eMSCs for clinical use need to be comprehensively evaluated in future studies.

## Supplementary Information

**Additional** **file** **1.  Supplementary Tables. Table S1. **Characteristics of endometrial cancer patients for EC organoid establishment.** Table S2. **Characteristics of samples for isolation of MSCs.** Table S3. **Primers for siRNAs.** Table S4. **Primers for qRT-PCR.

**Additional** **file** **2.** **Figure** **S1**. The identification of MSCs. **A**. Flow cytometry showed that MSC-specific markers CD73, CD90, and CD105 were expressed in three types of MSCs. Hematopoietic stem cell markers CD45 were not expressed in three types of MSCs. **B.** *In vitro* differentiation assays of AD-MSCs, UC-MSCs, and eMSCs. MSCs were induced to differentiate toward adipogenic lineage and verified by Oil Red O, osteogenic lineage and verified by von Kossa staining after induction, and chondrogenic lineage and verified by Alcian Blue staining. **C**. Flow cytometry showed positive expression of MSC markers CD73, CD90, and CD105 in eMSCs at passage 8. Hematopoietic stem cell markers CD45 were not expressed in eMSCs at passage 8.

**Additional** **file** **3.** **Figure** **S2**. The immunomodulatory effect of MSCs. Murine splenocytes were treated with CM from different MSCs or NM for 36 h in the LPS-stimulated condition, then were evaluated with immunophenotype characterization by flow cytometry. **A-B**. CM obtained from eMSCs had little effect on the proportion of CD4^+^/CD3^+^T cells and CD8^+^/CD3^+^T cells in the LPS-stimulated condition, while CM from AD-MSCs and UC-MSCs decreased the proportion of CD4^+^/CD3^+^T cells and CD8^+^/CD3^+^T cells. **C-E**. CM derived from the eMSCs did not influence the secretion of GzmB, INF-γ and TNF-α, CM from AD-MSCs and UC-MSCs slightly affected the level of GzmB and TNF-α. NM, normal medium; CM, conditioned medium; LPS, Lipopolysaccharides; Granzyme B. Data are representative of three independent experiments, and were analyzed by unpaired *t*-test. ns, not significant; *, P < 0.05; **, P < 0.01; ***, P < 0.001.

**Additional** **file** **4.** **Figure** **S3**. CM of eMSCs significantly inhibited EC cell viability. **A**. The inhibitory effect of CM from eMSCs on EC cells gradually and significantly increased with the increase of CM proportion. EC cells were seeded into 96-well plates at a density of 3000 cells per well and then treated with CM with different proportionfor 48 h and CCK-8 was used to measure EC cell proliferation level. **B.** CM derived from eMSCs showed the most significant anti-proliferative effect on EC cells, compared to AD-MSCs and UC-MSCs. HEC-1A cells were seeded at 1000 cells per well into 96-well plates and treated with NM or 100% CM derived from the three MSCs for indicated time period. CM was changed every 48 h during the experiment. Cell viability was measured by CCK-8. NM, normal medium; CM, conditioned medium; ns, not significant; Data were analyzed by ratio *t*-testand unpaired *t*-test. Green, red, and blue asteriskmeant P value between the NM and CM-eMSCs, CM-UC-MSCs, and CM-AD-MSCs respectively. *, P < 0.05; **, P < 0.01; ***, P < 0.001.

**Additional** **file** **5.** **Figure** **S4.** IHC staining in xenograft tumors. IHC staining images for DKK1and β-cateninin xenograft tumors in control group and eMSCs group. The percentage of β-catenin-positive area and DKK1-positive area were calculated by ImageJ. Original magnification, 40×; Scale bar, 50μm. Data were analyzed by ratio *t*-test. *, P < 0.05; **, P < 0.01.

**Additional** **file** **6.** **Figure** **S5**. DKK1 was an important factor that mediated eMSCs-induced Wnt/β-catenin signaling inhibition. **A.** DKK1 was silenced by si-DKK1 in eMSCs. DKK1 down-regulation in eMSCs was determined by western blotting. **B.** Decreased proliferation of EC organoids induced by CM of eMSCs was rescued in CM of eMSCs-siDKK1. EC organoids were treated with indicated CM for 96 h and then cell viability was assessed by three-dimensional cell viability assa**y.** EC-case 2 organoid line was used. NM, normal medium; CM, conditioned medium. The blots of DKK1 and GAPDH were all croppedand full-length blots were presented in **Supplementary** **Figure** **6**. Data were analyzed by ratio *t*-test. **, P < 0.01.

**Additional** **file** **7. Figure S6. **Full-length blots of  Western blotting analysis.

**Additional** **file** **8. **Supplementary Materials and Methods. Identification of MSCs and Immunomodulatory potential assay of MSCs.

**Additional** **file** **9. **Raw data of cell viability experiments.

## Data Availability

All datasets used and/or analyzed during the current study are available from the corresponding author upon reasonable request.

## Abbreviations

AD-MSCs: Adipose-derived MSCs
CCK-8: Cell Counting Kit-8
CK7: Cytokeratin 7
CM: Conditional medium
CR: Complete response
DKK1: Dickkopf-related protein 1
EC: Endometrial cancer
ERα: Estrogen receptor α
eMSCs: Endometrium-derived MSCs
H&E: Hematoxylin and eosin
IF: Immunofluorescence
LPS: Lipopolysaccharides
MPA: Medroxyprogesterone acetate
MSCs: Mesenchymal stem cells
NM: Normal medium
PR: Progesterone receptor
qRT-PCR: Quantificational real-time polymerase chain reaction
SD: Standard deviation
UC-MSCs: Umbilical cord-derived MSCs
