# Supplementary material for: Endometrium-derived mesenchymal stem cells suppress progression of endometrial cancer via the DKK1-Wnt/β-catenin signaling pathway
Source: Stem Cell Res Ther. 2023 Jun 7;14:159. doi: 10.1186/s13287-023-03387-4 (PMC10249217; doi:10.1186/s13287-023-03387-4)
Supplement: Supplementary file 1 — Additional file 1. Supplementary Tables. Table S1. Characteristics of endometrial cancer patients for EC organoid establishment. Table S2. Characteristics of samples for isolation of MSCs. Table S3. Primers for siRNAs. Table S4. Primers for qRT-PCR. [file 13287_2023_3387_MOESM1_ESM.docx]

**Supplementary Table S1. Characteristics of endometrial cancer patients for EC organoid establishment**

| Patients | Age  (year)^a^ | Hormone usage | Menstruation | BMI（kg/m^^2^） | Diagnosis | Tumor size(cm^^3^) | Stage | Grade | ERα | PR | Viable organoids |
| --- | --- | --- | --- | --- | --- | --- | --- | --- | --- | --- | --- |
| EC-case 1 | 50-59 | No | Menopause | 21.20 | Endometrioid endometrial cancer | 0.5x0.5x3.0 | IA | G1 | +, 40% | +,30% | yes |
| EC-case 2 | 50-59 | No | Pre-menopause | 21.78 | Endometrioid endometrial cancer | 1.0x0.8x3.0 | IA | G1 | +, 90% | +, 90% | yes |
| EC-case 3 | 60-69 | No | Menopause | 30.82 | Endometrioid endometrial cancer | 2.0x2.0x1.0 | IA | G1 | +, 80% | +, 40% | yes |

+: positive expression. ^a^ Range

**Supplementary Table S2. Characteristics of samples for isolation of MSCs**

| MSCs | Age  (year) ^a^ | Hormone usage | Menstruation | BMI（kg/m^^2^） | Menstrual phase | Diagnosis | Viable MSCs |
| --- | --- | --- | --- | --- | --- | --- | --- |
| eMSCs-case 1 | 50-59 | No | Pre-menopause | 27.10 | Proliferative phase | Leiomyoma | yes |
| eMSCs-case 2 | 30-39 | No | Pre-menopause | 28.83 | Secretory phase | Cervical intraepithelial neoplasia | yes |
| eMSCs-case 3 | 40-49 | No | Pre-menopause | 26.50 | Secretory phase | Cervical intraepithelial neoplasia | yes |

^a^ Range

**Supplementary Table S3. Primers for siRNAs**

| siRNA | Primer Sequence | |
| --- | --- | --- |
| DKK1-siRNA-1 | sense（5'-3'） | GCCGGAUACAGAAAGAUCATT |
|  | antisense（5'-3'） | UGAUCUUUCUGUAUCCGGCTT |
| DKK1-siRNA-2 | sense（5'-3'） | GUACCAAGCAUAGGAGAAATT |
|  | antisense（5'-3'） | UUUCUCCUAUGCUUGGUACTT |
| DKK1-siRNA-3 | sense（5'-3'） | GGAAUAAGUACCAGACCAUTT |
|  | antisense（5'-3'） | AUGGUCUGGUACUUAUUCCTT |
| siMOCK | sense（5'-3'） | UUCUCCGAACGUGUCACGUTT |
|  | antisense（5'-3'） | ACGUGACACGUUCGGAGAATT |

**Supplementary Table S4. Primers for qRT-PCR**

| Genes | Primer Sequence | | Annealing temperature (℃) |
| --- | --- | --- | --- |
| GAPDH | Forward | 5’-AACGGATTTGGTCGTATTG-3’ | 60 |
|  | Reverse | 5’-GGAAGATGGTGATGGGATT-3’ |  |
| ALDH1 | Forward | 5’-GACAATGCTGTTGAATTTGCAC-3’ | 60 |
|  | Reverse | 5’-AAGGATATACTTCTTAGCCCGC-3’ |  |
| BMI1 | Forward | 5’-CAAGACCAGACCACTACTGAAT-3’ | 60 |
|  | Reverse | 5’-TATCTTCATCTGCAACCTCTCC-3’ |  |
| NANOG | Forward | 5’-CCCAAAGGCAAACAACCCACTTCT-3’ | 60 |
|  | Reverse | 5’-AGCTGGGTGGAAGAGAACACAGTT-3’ |  |
| AXIN2 | Forward | 5’-CTGGCAACTCAGTAACAGCC-3’ | 60 |
|  | Reverse | 5’-GCCTGGTGTTGGAAGAGACA-3’ |  |
| C-MYC | Forward | 5’-CGACGAGACCTTCATCAAAAAC-3’ | 60 |
|  | Reverse | 5’-CTTCTCTGAGACGAGCTTGG-3’ |  |
| LGR5 | Forward | 5’-CTCCCAGGTCTGGTGTGTTG-3’ | 60 |
|  | Reverse | 5’-GAGGTCTAGGTAGGAGGTGAAG-3’ |  |
| DKK1 | Forward | 5’-CGGGCGGGAATAAGTACCAG-3’ | 60 |
|  | Reverse | 5’-CGAGACAGATTTGCACGCCT-3’ |  |
| WIF1 | Forward | 5’-TGAATTTTACCTGGCAAGCTG-3’ | 60 |
|  | Reverse | 5’-GGACATTGACGGTTGGATCT-3’ |  |
| SPRF-1 | Forward | 5’-GCTGGGGACTGCGCCTTTTGT-3’ | 60 |
|  | Reverse | 5’-CTGCCGCAAACTTCCAGGGACC-3’ |  |
