# Supplementary figures and images for: Endometrium-derived mesenchymal stem cells suppress progression of endometrial cancer via the DKK1-Wnt/β-catenin signaling pathway
Source: Stem Cell Res Ther. 2023 Jun 7;14:159. doi: 10.1186/s13287-023-03387-4 (PMC10249217; doi:10.1186/s13287-023-03387-4)

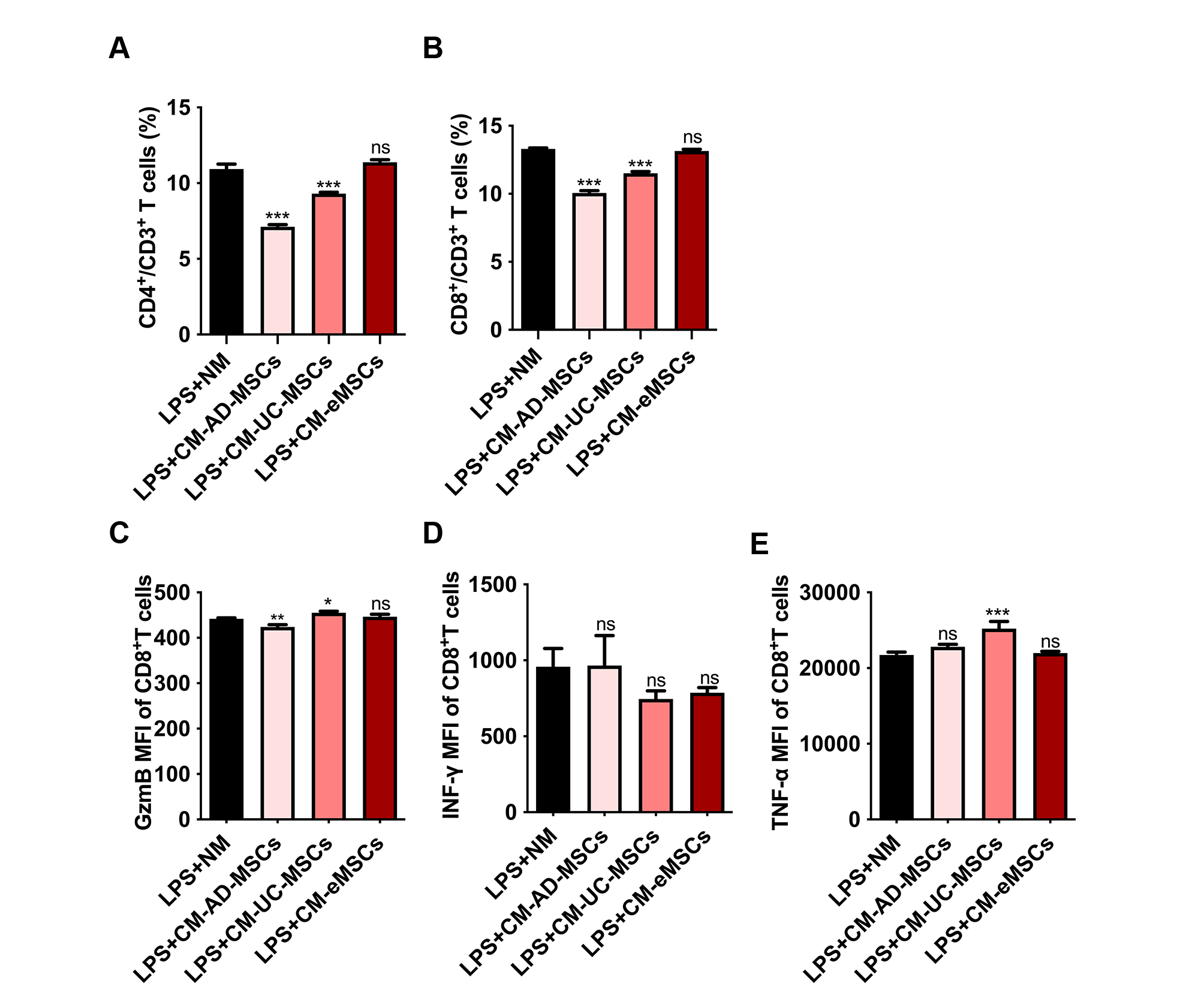

Supplement: Supplementary file 3 — Additional file 3. Figure S2. The immunomodulatory effect of MSCs. Murine splenocytes were treated with CM from different MSCs or NM for 36 h in the LPS-stimulated condition, then were evaluated with immunophenotype characterization by flow cytometry. A-B. CM obtained from eMSCs had little effect on the proportion of CD4+/CD3+T cells and CD8+/CD3+T cells in the LPS-stimulated condition, while CM from AD-MSCs and UC-MSCs decreased the proportion of CD4+/CD3+T cells and CD8+/CD3+T cells. C-E. CM derived from the eMSCs did not influence the secretion of GzmB, INF-γ and TNF-α, CM from AD-MSCs and UC-MSCs slightly affected the level of GzmB and TNF-α. NM, normal medium; CM, conditioned medium; LPS, Lipopolysaccharides; Granzyme B. Data are representative of three independent experiments, and were analyzed by unpaired t-test. ns, not significant; *, P < 0.05; **, P < 0.01; ***, P < 0.001. [file 13287_2023_3387_MOESM3_ESM.tif]

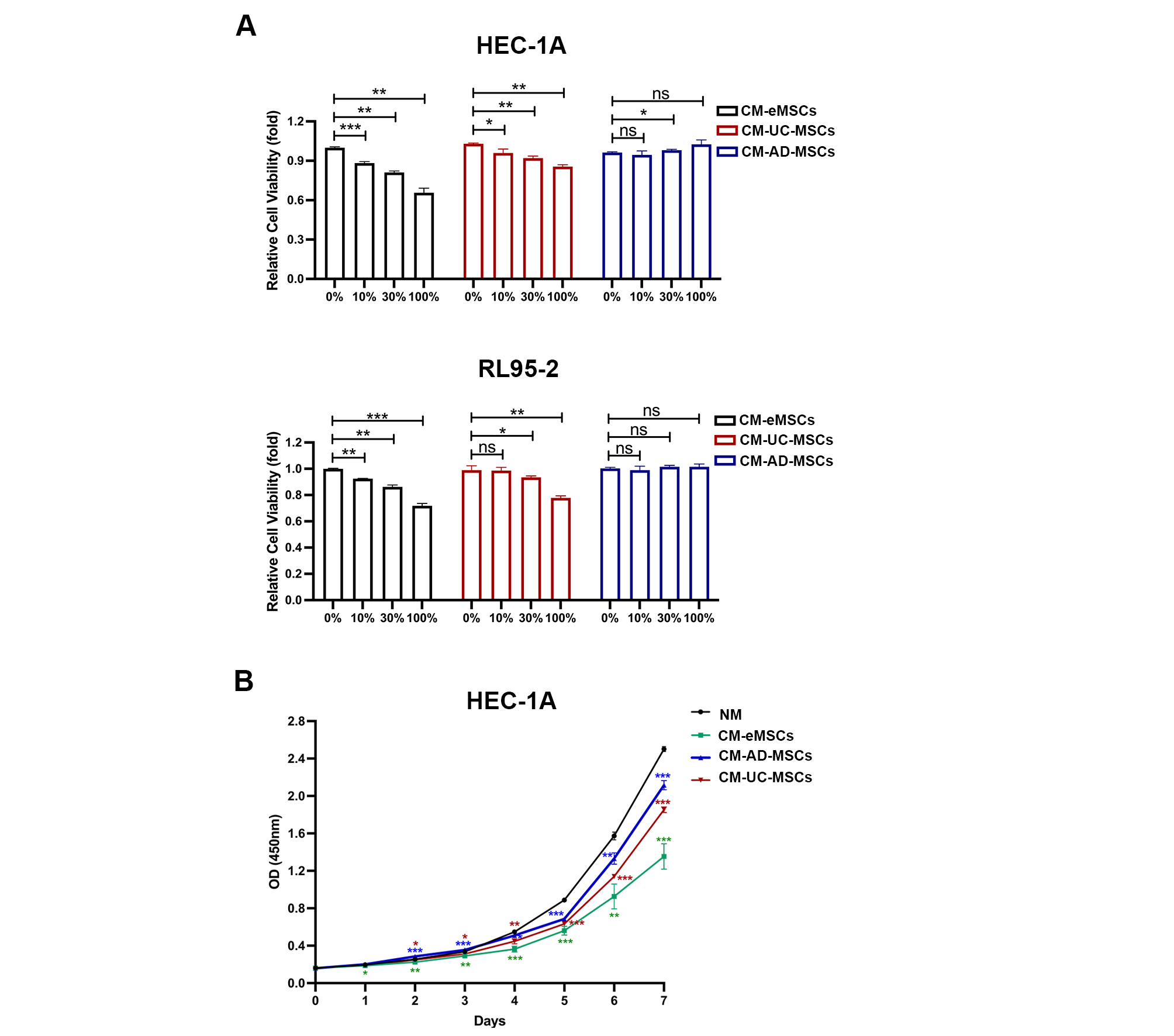

Supplement: Supplementary file 4 — Additional file 4. Figure S3. CM of eMSCs significantly inhibited EC cell viability. A. The inhibitory effect of CM from eMSCs on EC cells gradually and significantly increased with the increase of CM proportion. EC cells were seeded into 96-well plates at a density of 3000 cells per well and then treated with CM with different proportionfor 48 h and CCK-8 was used to measure EC cell proliferation level. B. CM derived from eMSCs showed the most significant anti-proliferative effect on EC cells, compared to AD-MSCs and UC-MSCs. HEC-1A cells were seeded at 1000 cells per well into 96-well plates and treated with NM or 100% CM derived from the three MSCs for indicated time period. CM was changed every 48 h during the experiment. Cell viability was measured by CCK-8. NM, normal medium; CM, conditioned medium; ns, not significant; Data were analyzed by ratio t-testand unpaired t-test. Green, red, and blue asteriskmeant P value between the NM and CM-eMSCs, CM-UC-MSCs, and CM-AD-MSCs respectively. *, P < 0.05; **, P < 0.01; ***, P < 0.001. [file 13287_2023_3387_MOESM4_ESM.tif]

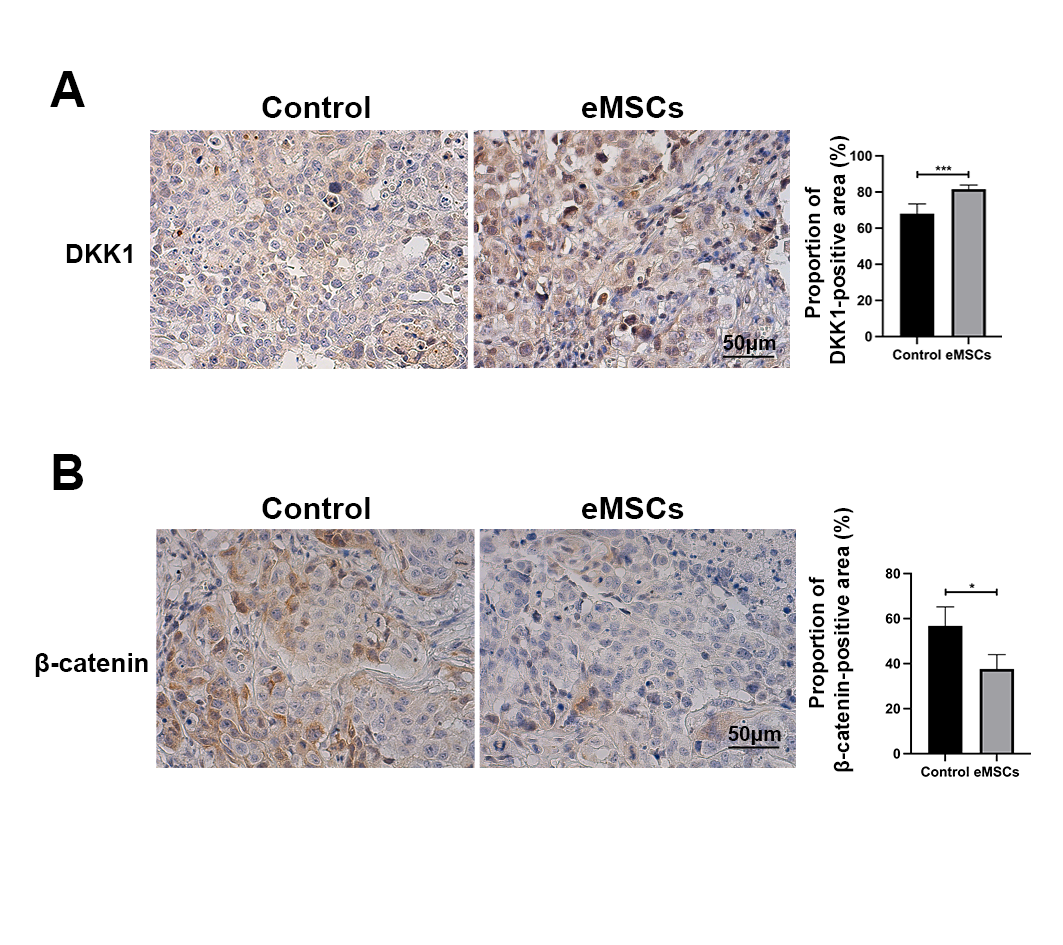

Supplement: Supplementary file 5 — Additional file 5. Figure S4. IHC staining in xenograft tumors. IHC staining images for DKK1and β-cateninin xenograft tumors in control group and eMSCs group. The percentage of β-catenin-positive area and DKK1-positive area were calculated by ImageJ. Original magnification, 40×; Scale bar, 50μm. Data were analyzed by ratio t-test. *, P < 0.05; **, P < 0.01. [file 13287_2023_3387_MOESM5_ESM.tif]

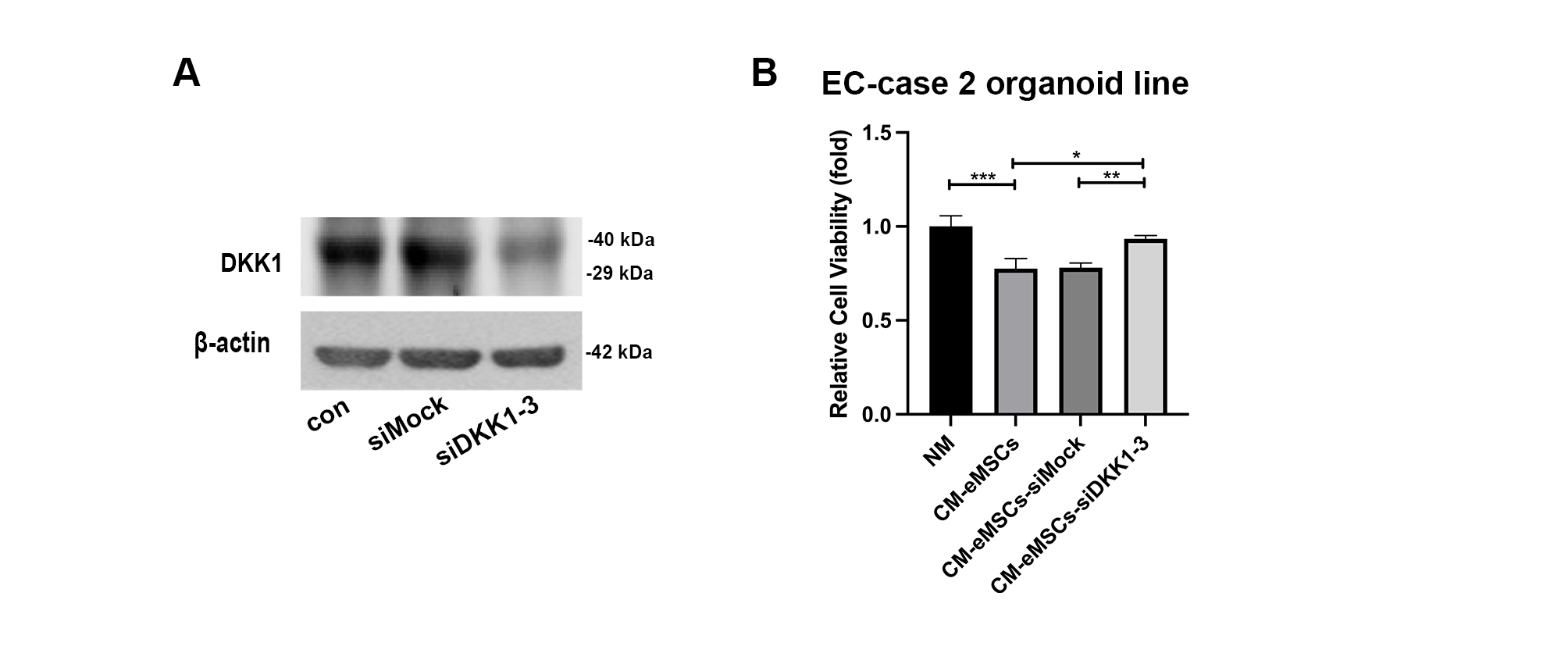

Supplement: Supplementary file 6 — Additional file 6. Figure S5. DKK1 was an important factor that mediated eMSCs-induced Wnt/β-catenin signaling inhibition. A. DKK1 was silenced by si-DKK1 in eMSCs. DKK1 down-regulation in eMSCs was determined by western blotting. B. Decreased proliferation of EC organoids induced by CM of eMSCs was rescued in CM of eMSCs-siDKK1. EC organoids were treated with indicated CM for 96 h and then cell viability was assessed by three-dimensional cell viability assay. EC-case 2 organoid line was used. NM, normal medium; CM, conditioned medium. The blots of DKK1 and GAPDH were all croppedand full-length blots were presented in Supplementary Figure 6. Data were analyzed by ratio t-test. **, P < 0.01. [file 13287_2023_3387_MOESM6_ESM.tif]
