# Supplementary material for: Endometrium-derived mesenchymal stem cells suppress progression of endometrial cancer via the DKK1-Wnt/β-catenin signaling pathway
Source: Stem Cell Res Ther. 2023 Jun 7;14:159. doi: 10.1186/s13287-023-03387-4 (PMC10249217; doi:10.1186/s13287-023-03387-4)

Supplementary Figure 6

Fig. 2B

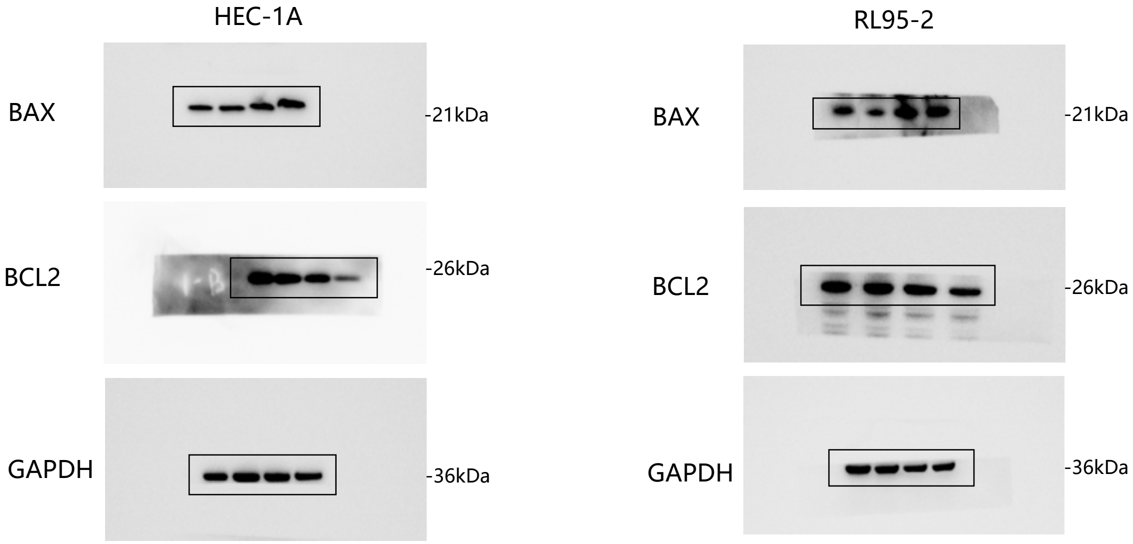

Fig. 4B

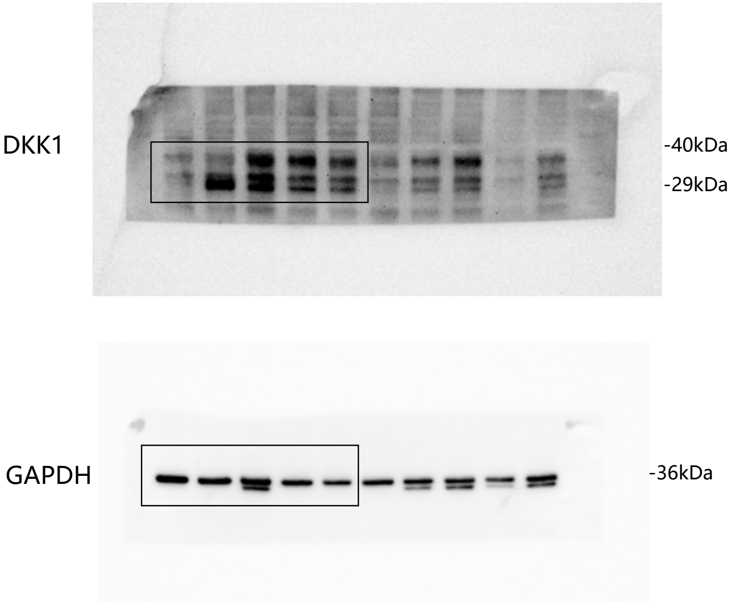

Fig. 4G

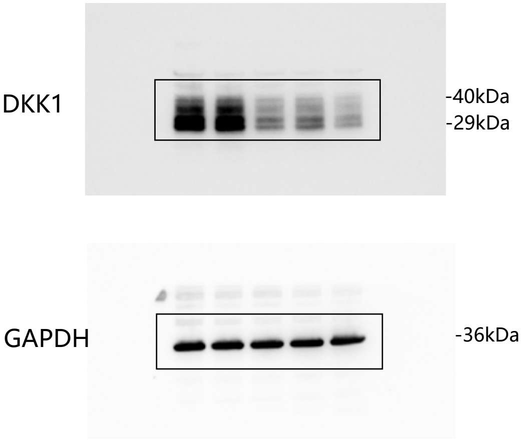

Fig. 5B

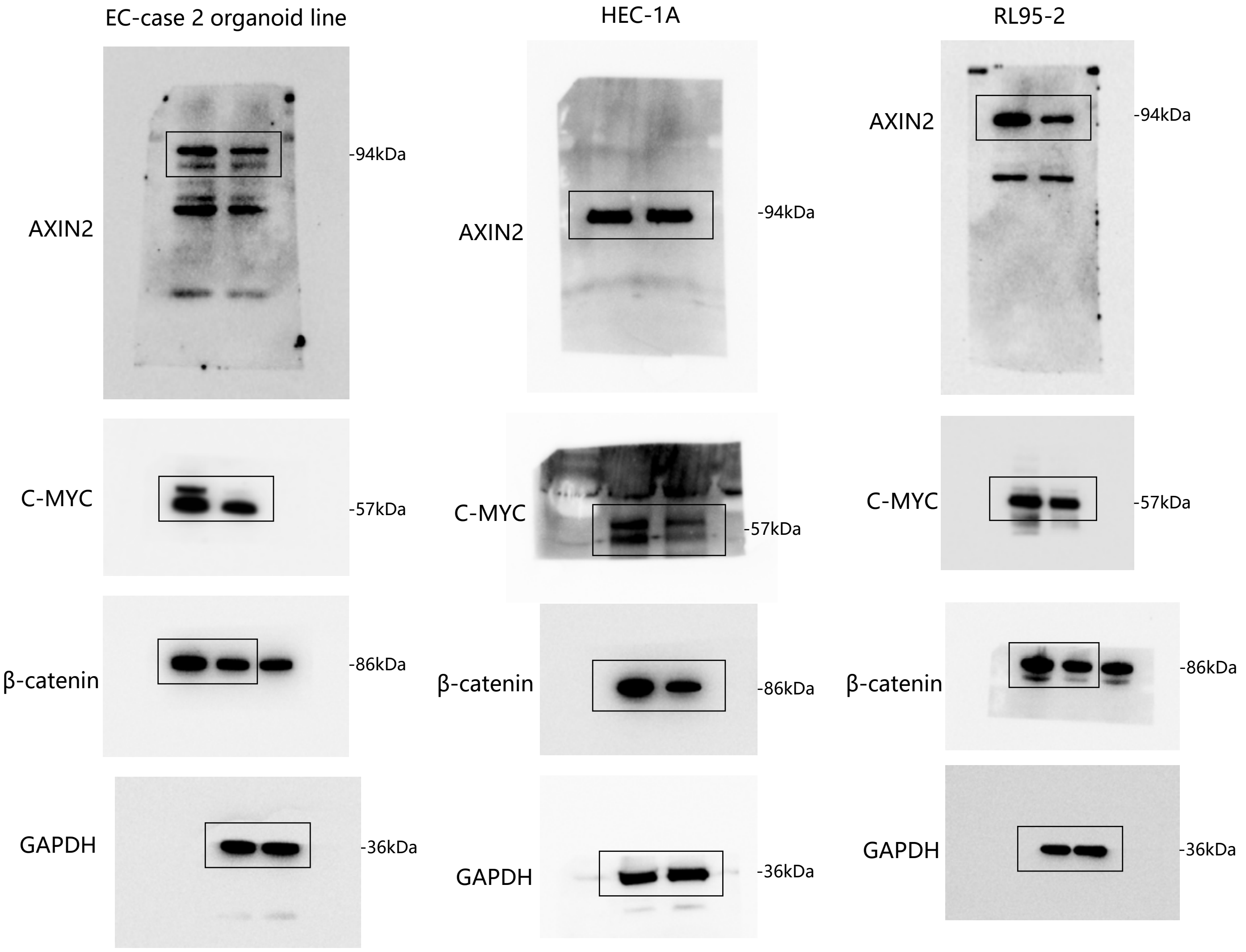

Fig. 5D HEC-1A

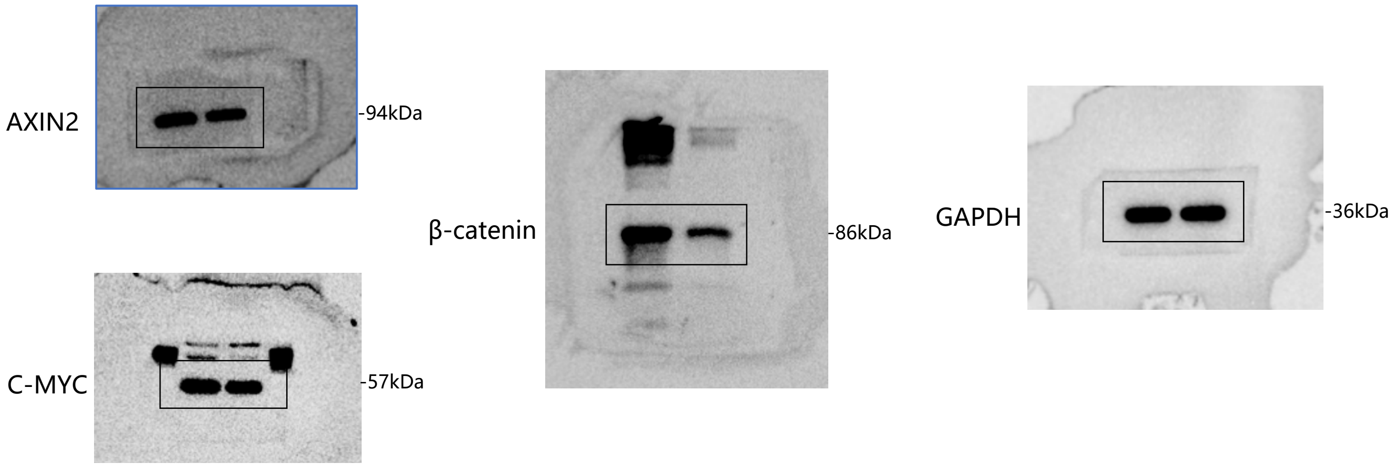

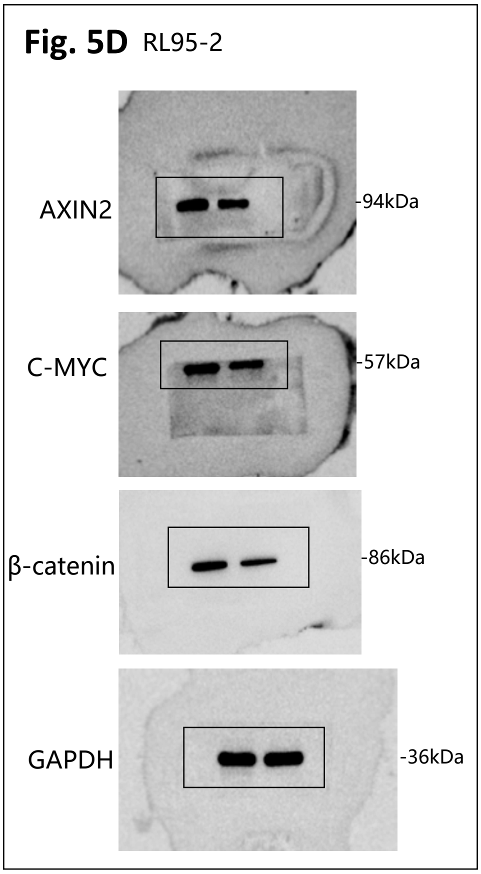
Fig. 5D
RL95-2
AXIN2
-94kDa
C-MYC
-57kDa
β-catenin
-86kDa
GAPDH
-36kDa

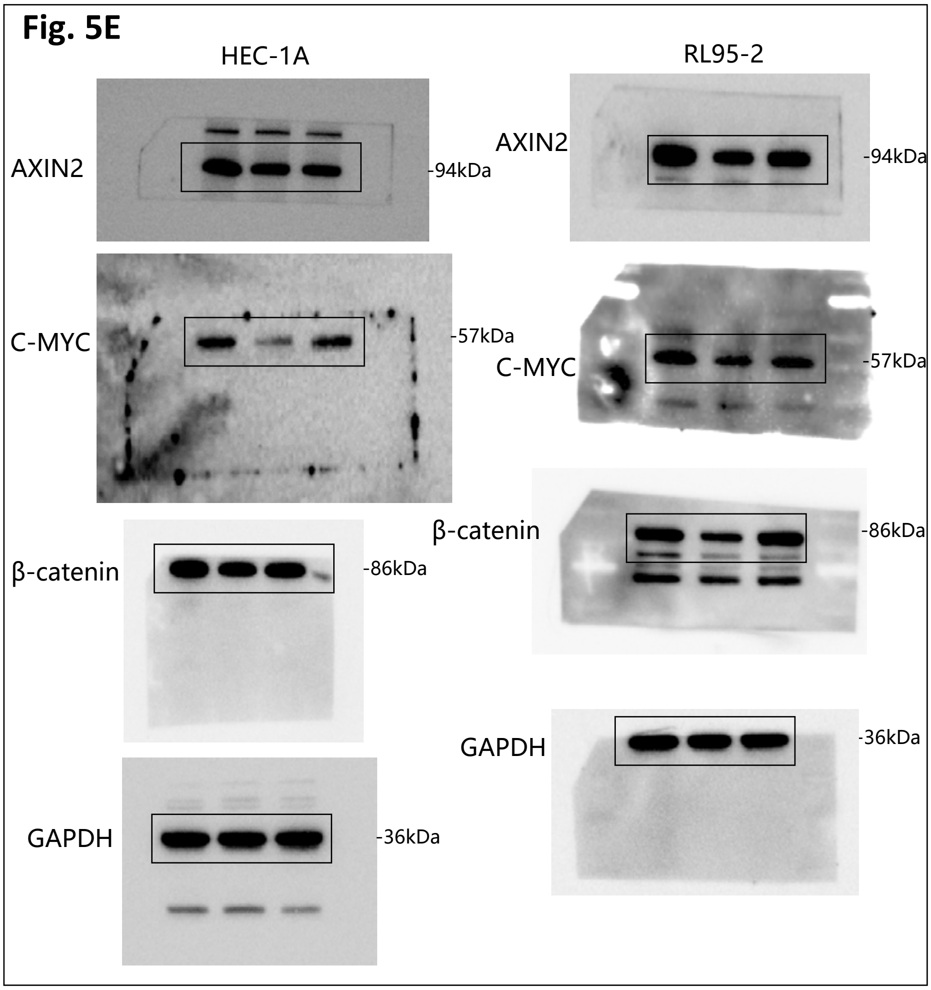
Fig. 5E
HEC-1A
RL95-2
AXIN2
-94kDa
C-MYC
-57kDa
β-catenin
-86kDa
GAPDH
-36kDa

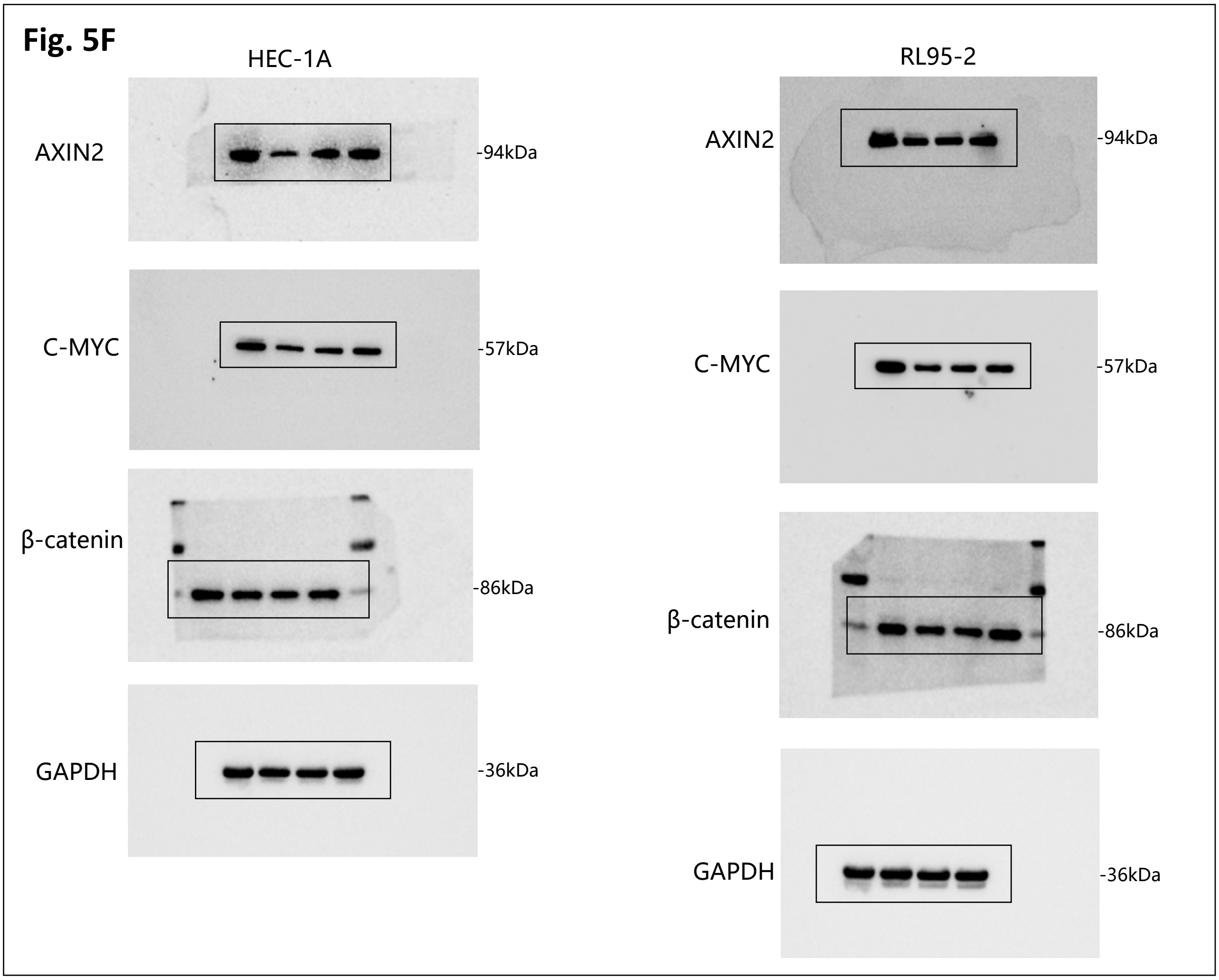
Fig. 5F
HEC-1A
RL95-2
AXIN2
-94kDa
C-MYC
-57kDa
β-catenin
-86kDa
GAPDH
-36kDa

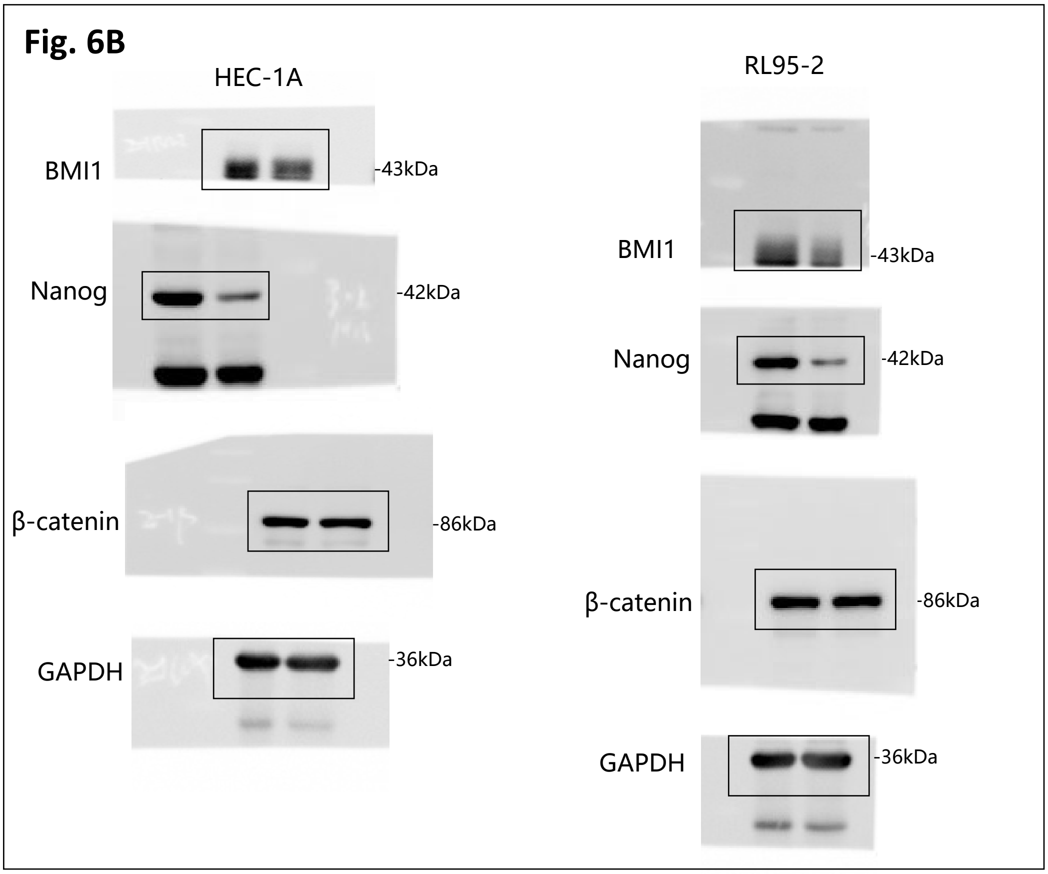
Fig. 6B
HEC-1A
RL95-2
BMI1
-43kDa
Nanog
-42kDa
β-catenin
-86kDa
GAPDH
-36kDa

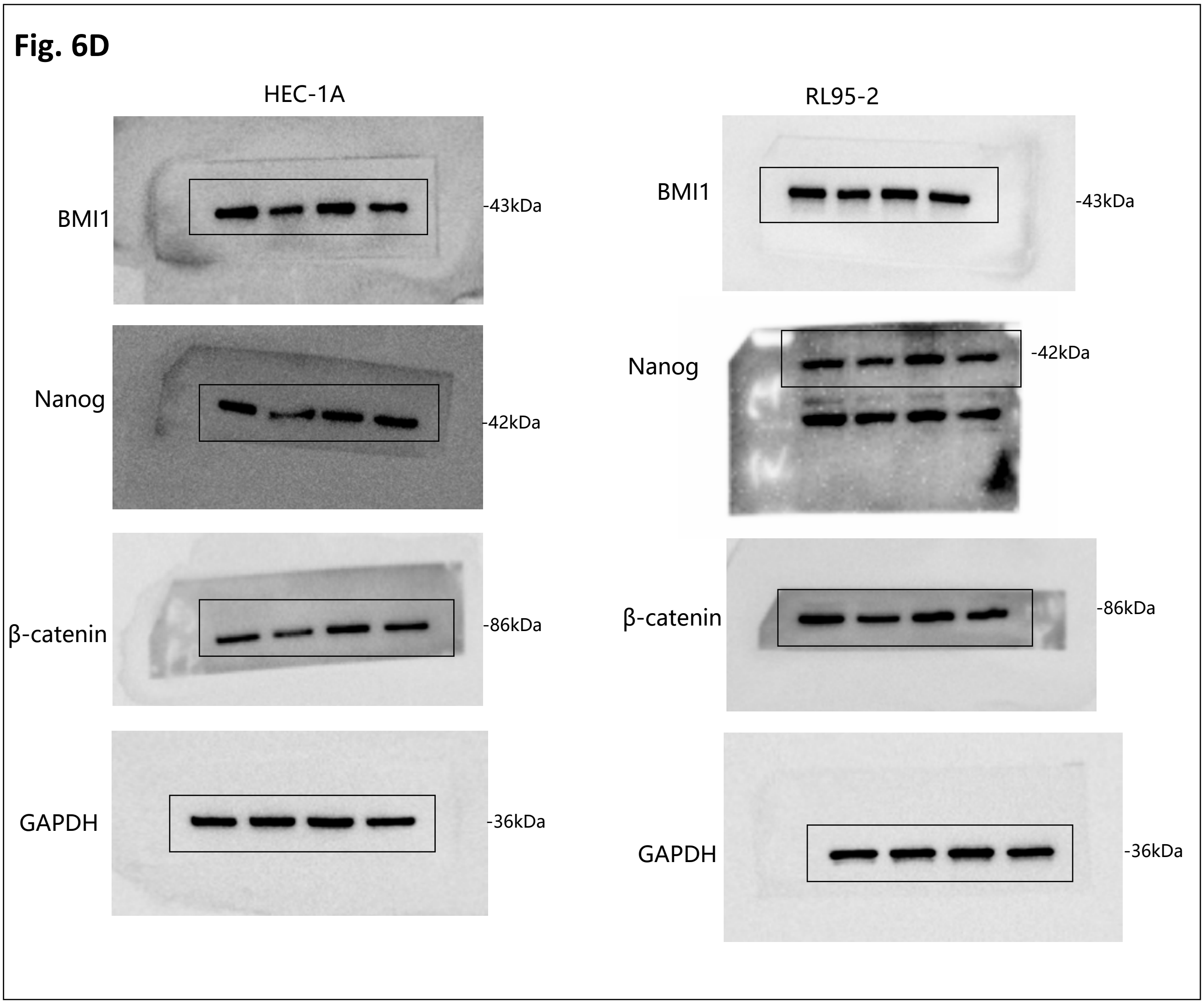
Fig. 6D
HEC-1A
RL95-2
BMI1
-43kDa
Nanog
-42kDa
β-catenin
-86kDa
GAPDH
-36kDa

Fig. 6E

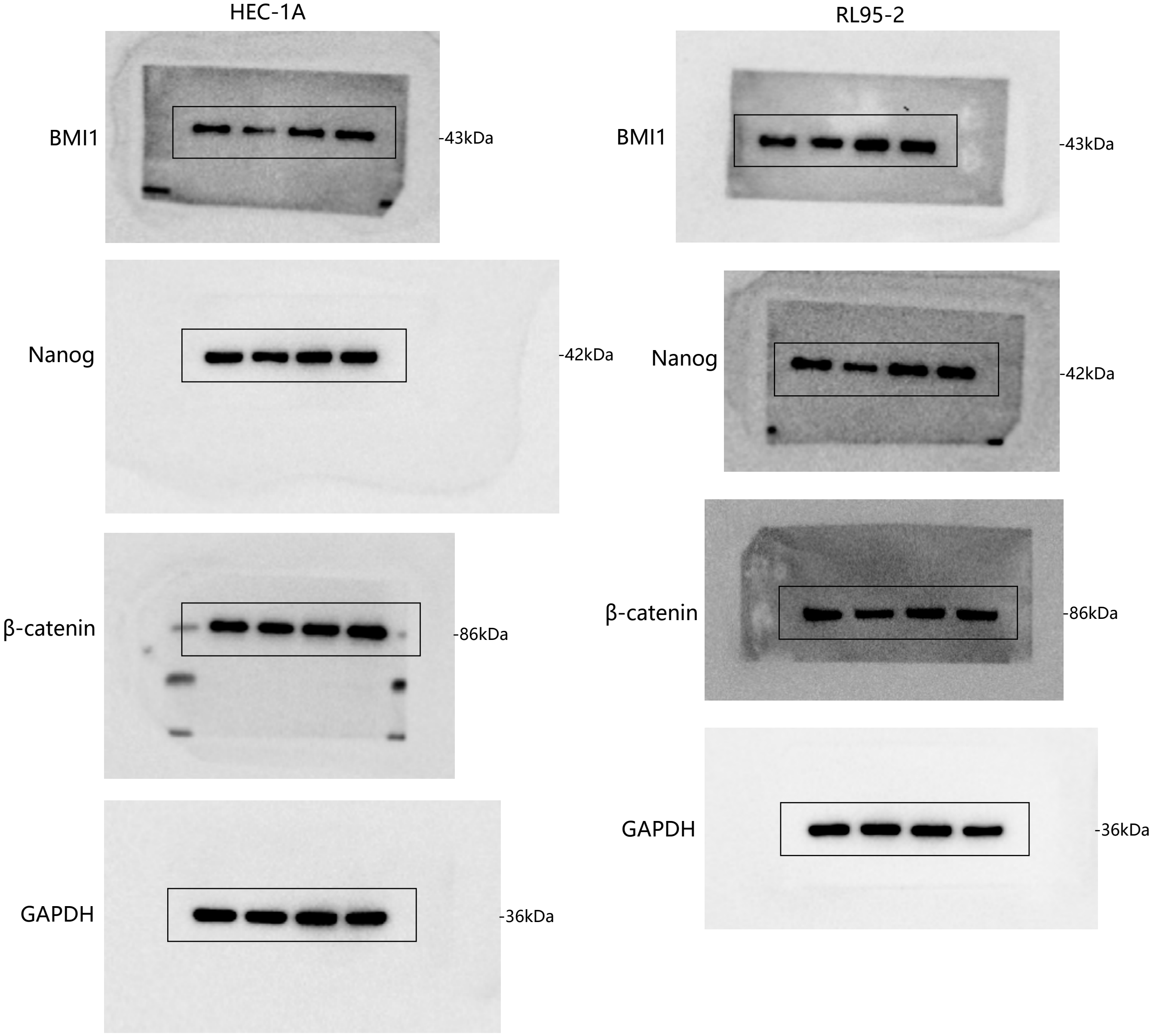

Fig. S5A

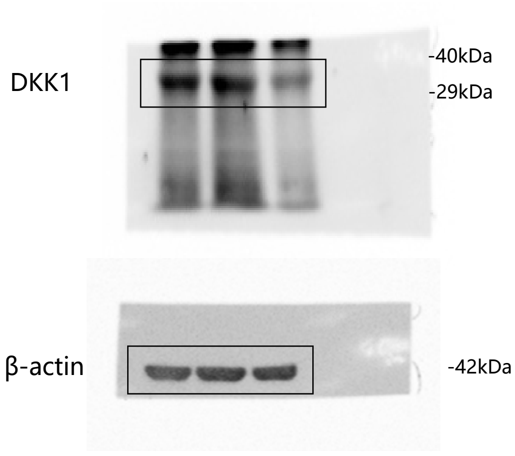

Supplement: Supplementary file 7 — Additional file 7. Figure S6. Full-length blots of Western blotting analysis. [file 13287_2023_3387_MOESM7_ESM.pdf]
