# Supplementary material for: Endometrium-derived mesenchymal stem cells suppress progression of endometrial cancer via the DKK1-Wnt/β-catenin signaling pathway
Source: Stem Cell Res Ther. 2023 Jun 7;14:159. doi: 10.1186/s13287-023-03387-4 (PMC10249217; doi:10.1186/s13287-023-03387-4)
Supplement: Supplementary file 8 — Additional file 8. Supplementary Materials and Methods. Identification of MSCs and Immunomodulatory potential assay of MSCs. [file 13287_2023_3387_MOESM8_ESM.doc]

**Supplementary Materials and Methods**

**Identification of MSCs**

MSCs were identified by flow cytometry using the following fluorescence-labeled antibodies: anti-CD45-PE, anti-CD73-PE, anti-CD90-PE, and anti-CD105-PE (all from BioLegend, USA), to confirm whether MSCs maintain the stemness between passage 5 and 8.

For *in vitro* differentiation assays of MSCs, AD-MSCs, UC-MSCs, and eMSCs were cultured with osteogenic, chondrogenic, and adipogenic induction medium for 3 weeks. After 3 weeks, MSCs were analyzed for osteogenesis, adipogenesis, and chondrogenesis by von Kossa staining, Oil Red O staining, and Alcian Blue staining, respectively.

**Immunomodulatory potential assay of MSCs**

Adult female C57BL/6 mice were sacrificed and spleen tissues were collected. Murine splenocytes were isolated by filtrating through 70 μm sieve (Corning, USA) and followed by red blood cell lysis solution (Biolegend, USA) to remove the red blood cells. Murine splenocytes were treated with 10 μg/mL Lipopolysaccharides (LPS) (Sigma Aldrich, USA) and CM from different MSCs or NM for 36 h, followed by flow cytometry analysis (Beckman). [Brilliant Violet 510™ anti-mouse CD45](http://bioec.cn/product/3_799717/Biolegend_103138_Brilliant Violet 510™ anti-mouse CD45_50 ug), [APC/Cyanine7 anti-mouse CD3](http://bioec.cn/product/3_77323/Biolegend_100222_APC_Cyanine7 anti-mouse CD3_100 ug), [PE anti-mouse CD4](http://bioec.cn/product/3_21620/Biolegend_100408_PE anti-mouse CD4_200 ug), [PE/Cyanine7 anti-mouse CD8a](http://bioec.cn/product/3_21718/Biolegend_100722_PE_Cyanine7 anti-mouse CD8a_100 ug), [FITC anti-human/mouse Granzyme B (GzmB) Recombinant](http://bioec.cn/product/3_1686722/Biolegend_372206_FITC anti-human_mouse Granzyme B Recombinant_100 tests), [PE anti-mouse IFN-γ](http://bioec.cn/product/3_28743/Biolegend_505808_PE anti-mouse IFN-γ_100 ug) and [Brilliant Violet 421™ anti-mouse TNF-α](http://bioec.cn/product/3_778497/Biolegend_506328_Brilliant Violet 421™ anti-mouse TNF-α_50 ug) were purchased from Biolegend.
